# Ethnopharmacology

**Published:** 2008-10

**101**

**Role of unripe *Carica papaya* linn. fruits on oxidative stress and hepatic glycogen content in experimentally induced diabetes in rats**

Sharma A, Srivastava DN, Akarte A, Deshmukh P, Parihar G;

**B. R. Nahata College of Pharmacy, Mandsaur - 458001, M.P., India.**

**Objective:**

The generation of ROS leading to oxidative stress and lipid peroxidation are responsible for late complications of diabetes mellitus. In present study, we examined possible protective effect of aqueous extract of unripe fruit of *Carica papaya* Linn. (AUC) in experimental alloxan induced diabetes in rats.

**Materials and Methods::**

The effects of AUC on fasting blood glucose level (FBGL), oxidative stress parameters such as malondialdehyde (MDA) levels, advanced oxidation protein products (AOPP) and glutathione (GSH) in liver homogenates, terminal hepatic glycogen content and serum biochemical parameters were observed. To induce diabetes alloxan was injected i.p. at a single dose of 150 mg/kg. AUC (400 mg/ kg/ day p.o.) was administered to a group of diabetic rats (n=6) for 11 consecutive days and the observed data was compared with glibenclamide (10mg/ kg/ day p.o.) (n=6).

**Results and Discussion:**

Administration of AUC caused a reduction of 38.67 % FBGL from 155.7±1.6 mg/dl to 95.5±1.0 mg/dl (*P*< 0.001 versus diabetic control), MDA from 22.53±0.14 nmole/ mg protein to 5.5±0.34 nmole/mg protein (*P*<0.001 versus diabetic control), AOPP from 0.80±0.05 nmole/ mg protein to 0.59±0.04 nmole/ mg protein (*P*<0.05 versus diabetic control). AUC decreased the oxidative stress by increasing GSH levels from 6.4±0.1 mg/100 g tissue to 7.53±0.02 mg/100 g tissue (*P*<0.05 versus diabetic control) and increased terminal hepatic glycogen from 3.29±1.4 mg/g to 8.78±0.015 mg/g (*P*<0.05 versus diabetic control). AUC caused significant improvement in the kidney, liver and lipid function.

**Conclusion:**

Results of present study suggest that Carica papaya Linn. have a moderate antihyperglycemic effect and this may be attributed to its antioxidant potential.

**102**

**Antihistaminic, anti-inflammatory and mast cell stabilizing activity of ethanolic extract of *Solanum xanthocarpum* in experimental animals**

Parmar SK^1^, Gangwal A^1^, Mardia RB^2^, Dudhregia AV^1^, Sheth NR^1^

**^1^Saurashtra University, Rajkot, India; ^2^N. R. Vekaria Insitute of Pharmacy, Junagadh, India.**

Ethanolic extract of *Solanum xanthocarpum* Schrad. and Wendl. (ESX) (Family: Solanaceae) was investigated for antihistaminic and mast cell stabilizing activity in experimental animals. ESX was also evaluated for the anti-inflammatory activity using acute (carrageenan-induced paw oedema) and chronic (cotton pellet granuloma) models of inflammation in rats. The extract was found to possess significant antihistaminic action in histamine-induced bronchocontraction in guinea pigs. This may be suggestive of direct blockage of histamine receptor by ESX. The mast cell degranulation induced by compound 48:80 was significantly reduced by ESX. The effect was comparable to the reference standard Sodium cromoglycate. ESX also showed potent anti-inflammatory activity on both acute and chronic models of inflammation. These results suggest that ESX inhibits release of inflammatory mediators from mast cells which may be one of the mechanisms for its anti-inflammatory activity. The toxicity studies report safety usage of the plant extract. The present study for the first time reveals antihistaminic, anti-inflammatory and mast cell stabilizing potentials of *S. Xanthocarpum*. The plant may be useful in asthma and other inflammatory disease.

**103**

**Evaluation of anti – inflammatory and membrane stabilizing property of Aya– Chedooram in swiss albino rats**

K Kairavi, Vadivelan R, Antony AS, Satish Kumar MN, Elango K, Sainadh AVSR, B Suresh

**JSS College Of Pharmacy, Ooty, India.**

Chendooram is a catergory of medicine with reddish colour and a powdery form. Aya Chendooram consists of iron fillings, vinegar and odena bark juice. The anti inflamatory activity of the drug was studied on rat red blood cells, mast cells stabilisation, carrageenin inudced and polyvinyl pyrolidine induced paw inflammation. Aya Chendooram showed significant membrane stabilizing activity on mast cells and rat red blood cells. The anti–inflammatory was activity of this drug also revealed that it possess good anti inflamatory activity against both in–vivo and in–vitro models. Significant anti - inflammatory effect was shown by the drug on carrageenin and polyvinyl pyrrolidine induced paw edema in rats in both 40mg/kg and 80mg/kg dose levels but not significant in 20mg/kg dose level. The anti inflammatory activity showed by the drug may be due to prostaglandin synthesis inhibition or mast cell stabilisation and is therefore essential for anti inflammatory activity.

**104**

**Acute oral toxicity study of methanol extract of *Woodfordia fruticosa* kurz. flower in rats**

Baravalia Y, Vaghasiya Y, Chanda S

**Saurashtra University, Rajkot, Gujarat, India. E-mail: yogesh_barvaliya@yahoo.co.in**

There is a high degree of concern regarding the secure use of plant extracts and, for this very reason, preclinical and clinic toxicological evaluation of these extracts are needed. With the aim to assure the safety of the extract, our purpose was to investigate the acute toxicity of the methanolic extract of *Woodfordia fruticosa* flower. In this study, rats were administrated orally with single dosages of 450, 1800 and 3600 mg/kg body weight of *Woodfordia fruticosa* extract. Mortality, signs of toxicity, body weight, food and water consumption, and gross behavioral changes in cage were observed for 14 days. After 14 days rats were sacrificed to take the organ weight. No mortality, signs of toxicity and abnormalities were observed during the experimental period. In addition, no significant differences were noticed in the body and organ weights between control and treated groups. These results show that the methanol extract of *Woodfordia fruticosa* is toxicologically safe by oral administration.

**105**

**Effect of extracts of *Phyllanthus niruri* stem on intestinal P-glycoprotein and cytochrome P450 enzymes**

Shroff KK,^1^ Harle UN,^1^ Gaikwad NJ^2^

**^1^AISSMS College of Pharmacy, Pune; ^2^Department of Pharmaceutical Sciences, R.T.M. Nagpur University, Nagpur, India.**

Cytochrome 450 enzymes and P-glycoprotein (Pgp) are the common targets for herbal drugs and therapeutic medicinal agents resulting in interaction. Herb-drug interactions are evidenced by many case reports resulting in precipitating fatal toxicity. *Phyllanthus niruri* is also known digestive, diuretic, antispasmodic, laxative, antihepatotoxic, hepatotonic, stomachic agent. The effect of ethanolic extract of *Phyllanthus niruri* stems on *in vitro* intestinal permeability of dexamethazone and oral clearance of different probes of cytochrome P450 was studied. Acute and chronic treatment of *Phyllanthus niruri* significantly (*P* < 0.01, F = 4.58, Df = 4) increased the absorptive permeability of dexamethazone as compared with that of saline and found even more than that of verapamil pre-treated groups and there by showed inhibition of Pgp. In studying the effect of *Phyllanthus niruri* extracts on metabolizing enzymes CYP 450; CYP1A2, CYP2C9, CYP2D6 were found to be not affected, but CYP3A, was found to be significant inhibited by hydro-alcoholic extracts of *Phyllanthus niruri*. It may conclude that *Phyllanthus niruri* showed negative modulation of Pgp and CYP3A which is indicative of its potential for modulation of absorption and metabolism of therapeutic drugs and importance towards pharmacokinetic herb-drug interaction.

**106**

**Interaction of *Tinospora cordifolia* with the decoction of cyclosporin**

Chaudhari PM^1^, Harle UN^2^, Gaikwad NJ^2^

**^1^AISSMS College of Pharmacy, Pune; ^2^Department of Pharmaceutical Sciences, R.T.M. Nagpur University, Nagpur, India.**

In order to investigate the effect of antidiabetic herb *Tinospora cordifolia* on cyclosporin absorption and disposition, rabbits were given cyclosporin (10 mg/kg) with or without decoction of bark. In a crossover design FPIA method was used to determine the blood concentration of cyclosporin. The decoction was characterized by their amritosides A, B, C and D contents. Results showed that the coadministration of *Tinospora cordifolia* significantly increased the C_max_ of cyclosporin by 68%. The AUC of cyclosporin was significantly elevated by 95% when coadministered with *Tinospora cordifolia*. Among the rabbits, 1/5 exhibits acute toxicity of cyclosporin after concomitant intake of *Tinospora cordifolia*. It indicates an interaction of amritosides with cyclosporin. It may conclude that the blood concentration of cyclosporin should be carefully monitored to avoid cyclosporin intoxication when coadministered with *Tinospora cordifolia* decoction.

**107**

**An appraisal of the antitumor activity of alcoholic extract of *Premna herbacea* roxb in Ehrlich’s ascitic carcinoma model**

Kumar Ravishankar, Pai KSR, Dhamija Isha, Setty MM, Manjula SN, Ramalingayya GV

**Manipal College of Pharmaceutical Sciences, Manipal University, Manipal-576104 Karnataka, India.**

Although Ayurvedic literature mentions the use of the root nodules of *Premna herbaceae* in ailments such as asthma, tumour and inflammation, scientific evaluation has so far not been done to verify these claims. The present study therefore was undertaken to find out the possible anticancer effect of the Root nodules of Premna herbacea Roxb. (Family:-Verbenaceae) in Ehrlich.s ascitic carcinoma (EAC) model. Ehrlich Ascitic Carcinoma was induced in Swiss albino mice by injecting 2.5 × 10^6^ cell/ml of tumor cell suspension by i.p. Anticancer activity of the alcoholic extract (250 and 500 mg/kg i.p) was compared with that of Cisplatin (3.5 mg/kg i.p) on parameters such as percentage increase in weight, median survival time, hematology and *in vitro* cytotoxic effect of extract on EAC cell by tryphan blue exclusion assay. Alcoholic extract of *Premna herbacea* Roxb showed significant (*P*< 0.05) anticancer activity at different doses, in that it reduced the body weight, prolonged the life span of the tumor bearing animal. Besides, it also reversed the cancerassociated hematological changes in tumor-bearing animal.

**108**

**Antitumor activity of ethyl acetate extracts of *Premna herbacea* roxb in ehrlich’s ascitic carcinoma model**

Pai KSR, Dhamija Isha, Setty MM, Manjula SN

**Manipal College of Pharmaceutical Sciences, Manipal University, Manipal-576104 Karnataka, India.**

Root nodules of *Premna herbacea* Roxb. (Family:-Verbenaceae) are used in traditional medicine for the treatment of various ailments such as asthma, tumors, inflammation etc. However, root nodules of Premna herbacea Roxb, have not been worked for possible anticancer activity in EAC model. Thus, the present study has been undertaken to study, if the ethyl acetate extract of the title plant has got any anticancer activity in EAC model. Ehrlich Ascitic Carcinoma was induced in Swiss albino mice by injecting 2.5 × 106 cell/ml of tumor cell suspension i.p. Anticancer activity of the ethyl acetate extract (250 and 500 mg/kg i.p) was compared with that of Cisplatin (3.5 mg/kg i.p) on parameters such as percentage increase in weight, median survival time, and in vitro cytotoxic effect of extract on EAC cell by tryphan blue exclusion assay. Ethyl acetate extract of *Premna herbacea* Roxb at different doses showed significant (*P*< 0.05) anticancer activity in that it reduced the body weight, prolonged the life span of the tumor bearing animal.

**109**

**Evaluation of linseed for immunomodulatory activity using pyrogallol induced immunosuppression in rats**

Sharma V, Vithlani D, Sathaye S S

**University Institute of Chemical Technology, Mumbai, India.**

**Introduction:**

Modulation of immune responses to alleviate the diseases has been of interest for many years. It is now recognized that immunomodulation could provide an alternative or complement to conventional chemotherapy for a variety of diseased conditions. Linseed is one of the oldest cultivated plants in India. Traditionally, it has been used as a laxative, expectorant, and demulcent for the symptomatic relief of mild gastrointestinal discomfort and as a poultice for symptomatic treatment of minor skin inflammations. Recent studies also indicate glucose lowering, lipid lowering, antitumor and estrogenic effects of linseed.

**Objective:**

The objective of the present work was to prepare extracts of linseed using different solvents and evaluate the same for immunomodulatory activity using pyrogallol induced rat model.

**Materials and Methods:**

Pyrogallol (50mg/kg, i.p) was administered to produce immunosuppression in Wistar rats (n=6/group) for 28 days and concomitantly treated with standard and test drug at three dose levels. Animals were challenged on the 14^th^ and 21^st^ day for immunological responses. Antibody titre determination, delayed type hypersensitivity reaction and *in vitro* phagocytosis studies were carried out on the 21^st^, 28^th^ and 29^th^ day. *In vivo* phagocytosis was also carried out using carbon clearance method.

**Results and Conclusion:**

Present study substantiates that linseed is a potent immunostimulant, significantly stimulating (*P*<0.01) both specific and non-specific immune responses in a dose dependent manner.

**110**

**Evaluation of hepatoprotective activity of ethanolic extract of bark of *Cassia fistula***

Vadnal PY^1^, Sakhare GR^1^, Patwardhan SK^1^, Singhai AK^2^, Somani RS^1^

**^1^Sinhgad College of Pharmacy, Pune, India; ^2^Dept. of Pharm. Sci., Dr. HS Gour University, Sagar, India.**

**Objective:**

*Cassia fistula* L., (Leguminoseae), a semi-wild Indian Laburnum (also known as the Golden Shower). In spite of the vast pharmacological activities of this plant, its protective effect on the liver toxicity has not been well documented. Hence, the present investigation was designed to evaluate the hepatoprotective activity of ethanolic extract of bark of *Cassia fistula*.

**Method:**

The ethanolic (95%) extract of bark of *Cassia fistula* (CFB) was prepared by maceration. Carbon tetrachloride with olive oil (1:1) (0.2ml/kg, i.p.) was administered for ten days used to induce hepatotoxicity. CFB (200 and 400 mg/kg, *p.o*.) and Silymarin (100 mg/kg p.o.) were administered for fourteen days. The carbon tetrachloride, Silymarin and the extract were administered concomitantly to the respective groups of animals. The hepatoprotective effect of the extract was evaluated by the assay of liver function biochemical parameters (serum transaminase ALT, AST, ALP, bilirubin, protein and triglycerides).

**Results:**

The results obtained showed that the toxic effect of carbon tetrachloride was controlled significantly with the treatment of CFB (200 and 400 mg/kg p.o.) by restoration of the levels of serum transaminase ALT, AST, ALP, bilirubin, protein and triglycerides.

**Conclusion:**

Thus, *Cassia fistula* bark possesses potential to protect the liver against carbon tetrachloride induced hepatotoxicity in rats.

**111**

**Evaluation of anti-inflammatory and membrane stabilizing property of ethanol root extract of *Rubus ellipticus* smith in albino rats**

Singh K, Vadivelan R, Shanish A, Satish Kumar MN, Elango K, Suresh

**J.S.S College of Pharmacy, Ooty, India.**

This study reports the anti-inflammatory and membrane stabilizing property of an ethanolic root extract of Rubus ellipticus (RE) Smith in rats. It belongs to Rosaceae family commonly known in India as Zardanchu or Hinsalu in Hindi and in English yellow Himalayan raspberry. The carrageenan-induced rat paw edema was utilized as a model for acute inflammatory and the probable mode by which Rubus ellipticus mediates its effects on inflammatory condition was studied on rat blood cells exposed to hypotonic solution. The results of the study revealed that the extract possesses anti-inflammatory activity. Rubus ellipticus significantly (*P*<0.01, *P*<0.001) reduced the oedema swelling induced by carrageenin in rats at both the dose levels of 250 and 500 mg/kg, while 125mg/kg did not show any significant activity. However, the extract did not exhibit membrane stabilizing property, as it failed to significantly (*P*<0.05) reduced the levels of haemolysis of RBC exposed to hypotonic solution. The acute toxicity studies of oral doses of ethanolic root extract in rats revealed that it has a high safety profile, as the extract was well tolerated by the animals. The results of the study suggest that the anti-inflammatory activity demonstrated by Rubus ellipticus may not be related to membrane stabilization.

**112**

**Protective effect of *Calotropis procera* latex against ethanol induced gastric ulcers**

Bharti S, Wahane VD, Kumar VL

**Department of Pharmacology, All India Institute of Medical Sciences, New Delhi, India.**

**Introduction:**

In traditional system of medicine a number of herbal preparations have been used for the treatment of peptic ulcers. **Calotropis procera** is one such plant that has been used for the treatment for gastrointestinal disorders. The chloroform extract of its roots has been reported to exhibit antiulcer properties. In view of this, in present study we have evaluated the antiulcer effect of latex of *Calotropis procera* in rat model.

**Method:**

Gastric ulcers were induced in rats by oral administration of 1ml absolute ethanol. The rats were sacrificed 1hr later and stomach was dissected out and examined for calculating ulcer index. Different doses of aqueous extracts (20 and 100mg/kg) and methanol extracts (10 and 50 mg/ kg) of dried latex of *Calotropis procera* and famotidine (20mg/kg) were orally administered 1hr prior to the administration of absolute ethanol. The parameters of oxidative stress were also measured in the gastric mucosal tissue.

**Results:**

Treatment of rats with aqueous extract and methanol extract of dried latex produced a marked reduction in ulcer index. The extent of inhibition was found to be 58.33% and 95.83% in case of aqueous extracts (20 and 100mg/ kg) and 83.33% and 81.25% in case of methanol extracts (10 and 50 mg/kg) respectively. The protection afforded by latex extracts was comparable to the standard antiulcer drug famotidine (62.5%). The protective effect was accompanied by normalization of levels of parameters of oxidative stress.

**Conclusion:**

Our study shows that latex of *Calotropis procera* has the potential to be used as an antiulcer agent.

**113**

**Anti-obese activity of *Butea monosperma* bark extract in diet induced obese rats**

Dixit P, Prakash T, Divakar Goli, Swamidasan R, Kamalesh DR, Chandrasekar SB

**Acharya and B.M. Reddy college of Pharmacy, Bangalore-560090, India.**

**Introduction:**

Obesity is chronic condition characterized by excess body fat that is quantified by the elevation in body weight of the patient. It is accepted that obesity result from disequilibrium between energy intake and expenditure and this condition has a large impact on several metabolic and chronic ailments including heart disease, arthritis, hyperlipidemia and NIDDM. To date, pharmacological treatments do not appear to be effective in producing sustained long-term weight loss. Therefore, future research is necessary to discover new drug therapies that can be used to reduce the prevalence of obesity. So that the present study was undertaken to evaluate ethanolic extract of *B.monosperma* bark (EEBM).

**Methods:**

Obesity was induced in albino rats by feeding cafeteria diet /atherogenic diet daily for 40 days to Group I to VI. Group I and II are referred as Negative and Positive control respectively. EEBM was administered in dose of 200, 400, 800mg/kg, p.o. to Group III, IV and V. The effect of extract on following parameters was recorded – body weight, food intake, body temperature, locomotor activity and biochemical parameters like serum glucose, cholesterol and triglyceride levels.

**Result:**

The EEBM treatment at 400 and 800-mg/kg caused significant reduction in body weight, food intake, serum glucose, lipid level and increase in locomotor activity and body temperature.

**Conclusion:**

The result of the present study concludes that EEBM showed anti-obese activity in cafeteria and atherogenic diet fed rats. The effect produced was comparable with that produced by standard anti-obese drug sibutramine.

**114**

**Effect of *Tectona grandis* linn. on dexamethasoneinduced insulin resistance in mice**

Ahire YS, Ghaisas MM, Navghare VV, Takawale AR, Zope VS, Tanwar MB, Deshpande AD

**Padm. Dr. D. Y. Patil Institute of Pharmaceutical Sciences and Research, Pimpri, Pune 411 018, India.**

The bark of *Tectona grandis* Linn. is traditionally used in the treatment of diabetes. The present study was undertaken to investigate the effect of ethanolic extract of bark of *Tectona grandis* Linn. (TG) in dexamethasone induced insulin resistance in mice. Mice were treated with prestandardised dose of dexamethasone for 22 days and effect on plasma blood glucose level, serum triglyceride level, glucose uptake in skeletal muscle, levels of hepatic antioxidant enzymes (GSH, SOD, Catalase, LPO), and body weight was observed. TG showed significant decrease in plasma glucose and serum triglyceride levels (*P*<0.01) and also stimulated glucose uptake in skeletal muscle. The levels of antioxidant enzymes GSH, SOD, and catalase were significantly increased (*P*<0.01) and there was significant decrease (*P*<0.01) in level of LPO indicating the antioxidant activity of *Tectona grandis*. Hence it can be concluded that *Tectona grandis* may prove to be effective in the treatment of type II diabetes mellitus owing to its ability to decrease insulin resistance.

**115**

**Antioxidant and hepatoprotective activity of *Justicia gendarussa* burm**

Krishna KL^1^, Patel Jagruti A^2^

**^1^JSS College of Pharmacy, SS Nagara, Mysore, India; ^2^Institute of Pharmacy, Nirma University, Ahmedabad, India.**

**Objective:**

To evaluate the *in vitro* antioxidant activity and *in vivo* hepatoprotective activity of methanolic extract of leaves of *Justicia gendarussa*.

**Methods:**

Methanolic extract of leaves of *J. gendarussa* (JME) was prepared by soxhlet extraction method. The antioxidant potency of JME was evaluated by using well established *in vitro* methods, such as DPPH radical scavenging, Hydroxyl radical (·OH) scavenging H_2_O_2_ scavenging and Ferric ion reduction activity. Also Total Phenolic Content (TPC) and flavonoid content (TFC) were determined. *In vivo* hepatoprotective activity was tested on CCl_4_ induced acute heaptototoxicity in rats at 3 different dose levels. The different biochemical estimations were carried out like SGOT, SGPT, total bilirubin, total proteins, GST, SOD, Catalase and peroxidase.

**Results:**

JME showed potent antioxidant activity *in vitro*. In each method IC_50_ values were determined and compared with different standards. IC_50_ values of JME in DPPH radical scavenging, Hydroxyl radical (·OH) scavenging, H_2_O_2_ scavenging and Ferric ion reduction activity were comparable with standards. JME protected the rats significantly when compared to control (CCl_4_ treated rats) in all the parameters. TPC and TFC were found to be 164.16+3.00 (mg GAE/g of extracts), 54.33+ 4.50 mg/g of quercetin.

**Conclusion:**

JME protected the rats by CCl_4_ injury which is a free radical mediated. JME also showed potent antioxidant activity *in vitro*. The hepatoprotective activity of JME might be due to its antioxidant activity, which is may be due to the total phenolic/flavonoid content of the extract. So it was concluded that total phenolic/flavonoids are responsible for its hepatoprotective activity.

**116**

**Pharmacological evaluation for anti-ulcer effect of *Cynodon dactylon* pers. Against gastric ulcers in rats**

Dhoke V, Mishra A, Vohra R, Ghosh R, Kadam VJ

**Bharati Vidyapeeth’s College of Pharmacy, C.B. D. Belapur, Navi Mumbai, India.**

**Introduction:**

Peptic ulcer occurs due to imbalance between offensive acid-pepsin secretions versus defensive (cytoprotective) mucosal resistance. Increased use of common NSAIDS like aspirin, indomethacin etc. causes damage by inhibiting biosynthesis of cytoprotective prostaglandins. These drugs cannot be used for long term in the treatment of chronic inflammatory conditions like rheumatoid arthritis with peptic ulcer. In this study, effect of 50% ethanolic extract of *Cynodon dactylon* Pers. on ulcerogenic potential of indomethacin was studied.

**Materials and Methods:**

50% ethanolic extract of *Cynodon dactylon* Pers. was administered in the dose of 300 and 600 mg/ kg orally 30 minutes prior to ulcer induction in Male Sprague-Dawley rats. The gastric ulcers were induced by oral administration of indomethacin. Famotidine was used as a reference standard drug.

**Results:**

The antiulcer activity was assessed by determining and comparing the ulcer index in the test drug group with that of the vehicle control group and standard group. Both the doses, 300 and 600 mg/ kg of test drug showed a protective effect on indomethacin- induced ulcers with 56.74% and 79.61% ulcer inhibition rate, respectively.

**Conclusion:**

These results suggested that *Cynodon dactylon* Pers. possesses anti- ulcer activity

**117**

**Phosphodiesterase inhibitors 9 (PDE9): potential therapeutic agents**

Sawant O, Ghosh R, Kadam VJ

**Bharati Vidyapeeth’s College of Pharmacy, CBD Belapur, Navi Mumbai, India.**

A phosphodiesterase inhibitor is a drug that blocks one or more of the five subtypes of the enzyme phosphodiesterase (PDE), therefore preventing the inactivation of the intracellular second messengers, (cAMP) and (cGMP). PDE9 is highly specific for cGMP and not inhibited by the nonselective phosphodiesterase inhibitor 3-isobutyl-1-methyl-xanthine (IBMX). BAY 73-6691 has been characterized *in vitro* as the first potent and selective inhibitor of phosphodiesterase 9 (PDE9), which is currently under preclinical development for the treatment of Alzheimer’s disease. BAY 73- 6691 improves learning and memory in rodents. Zardaverine (ZAR) and SCH 51866 are selective inhibitors and have recently been introduced as a potent bronchodilator and antiplateletantiproliferative agents respectively. Currently preclinical trials on PDE9 inhibitors for treating type 2 diabetes, metabolic syndrome, and cardiovascular disease are underway. Here we review the potential of PDE9 inhibitors as novel therapeutic agents for the treatment of a wide array of diseases.

**118**

**Bioassay-directed isolation of hypotensive alkaloid from *Holarrhena pubescens***

K Aftab^1^, A Mazhar^1^, SB Usmani, S Begum, BS Siddiqui

**^1^Department of Pharmacology and Therapeutics, Wah Medical College. Wah Cantt. H.E.J. Research Institute of Chemistry, University of Karachi, Karachi, Pakistan.**

*Holarrhena pubescens* belongs to the family Apocynacea, commonly known as “kurchi” is highly reputed in traditional medicine as a remedy for amoebic dysentery and other intestinal ailment. Bioassay-directed fractionation of the ethanolic extract of *Holarrhena pubescens* resulted in the isolation of steroidal alkaloids i.e. holamide and pubscinine. Holamide showed a three proton doublet at 1.45 (J=6.56 Hz) and two AB doubles at 3.17 and 3.00 each for on proton (J=12.06 Hz) in the 1H NMR spectrum suggested that it belongs to conanine series of alkaloid (A class of compound with the steroid nucleus and a five members heterocyclic ring with nitrogen). In contrast pubscinine showed one methyl at 1.28 while the doublet is missing a three proton singlet was observed at 2.28 due to a vinylic methyl indicated a double bond in the 18, 20 – epimino ring of the conanine series of alkaloids. In anaesthetized rats, the holamide and pubscinine caused a fall in blood pressure in a dose-dependent manner. Pretreatment of animals atropine completely abolished the hypotensive response of acetycholine; whereas hypotensive effect of holamide and pubscinine were not modified by atropine. Similarly acetylcholine produced contractile effect in guinea-pig ileum, which was antagonized by atropine, however both (holamide and pubscinine) failed to produced any stimulant response on guinea-pig ileum. These data indicate that the steroidal alkaloids i.e. Holamide and Pubscinine from *Holarrhena pubescens* mediated hypotensive response through a mechanism different to that of acetycholine.

**119**

**Antihyperglycemic and antihyperinsulinemic effect of aquous extract of *Aegle marmolos* leaf and *Encostemma littorale* herb in fructose fed rats**

Gohil TA^1^, Patel JK^1^, Vaghasiya JD^2^, Manek RA^2^

**^1^S. K. Patel College of Pharmaceutical Education and Research, Kherava, Mahesana, India, ^2^Smt, R. B. Patel Mahila Pharmacy College, Atkot, Gujarat, India**

**Introduction:**

Insulin resistance is a major contributor to the development of hyperglycemia in NIIDM patients. Aqueous extracts of *Aegle marmolos* and *Encostemma Littorale* has been shown to reduce hyperglycemia in diabetic animal models. Attempt has not been made to assess their effect on insulin resistance.

**Objective:**

This study was design to understand the effects aqueous extracts of *Aegle marmolos* and *Encostemma Littorale* on the Insulin resistance and metabolic parameters.

**Materials and Methods:**

Insulin resistance in wistar rat was induced by fructose rich diet (60% for 15 days). Treated groups have received fructose diet plus aqueous extracts *Aegle marmolos* (500 mg/kg/ day) and *Encostemma Littorale* (2gm/kg/day). Body weight, serum glucose, insulin, C-reactive protein and triglycerides levels were estimated.

**Results and Discussions:**

Fructose feeding for 15 days significantly increased serum glucose, insulin, C-reactive protein and triglycerides levels compared to control. Administration of aqueous extracts of *Aegle marmolos* and *Encostemma Littorale* for 15 days prevented hyperglycemia and hyperinsulinemia induced by a diet high in fructose.

**Conclusion:**

*Aegle marmolos* and *Encostemma Littorale* extracts might prove useful in the prevention of insulin resistance in non-diabetic state such as obesity and impaired glucose tolerance.

**120**

**Antiasthmatic potential of methanol fraction of aqueous extract of *Clerodendron phlomidis***

Vadnere GP^1^, Singhai AK^2^, Gaud RS^3^, Somani RS^4^

**^1^Smt.S.S.Patil College of Pharmacy, Chopda, India; ^2^University of Sagar, Sagar, India; ^3^NIIMS, Mumbai, India; ^4^Singhgad College of Pharmacy, Pune, India.**

**Introduction:**

In our previous study, dose dependent relaxant effect on histamine precontracted goat tracheal chain with significant decrease in eosinophilia, capillary permeability and protection of mast cells were demonstrated in mice. Pursuing the further investigation by activity guided fractionation of aqueous extract of *Clerodendron phlomidis* using benzene, ethyl acetate and methanol to screen the antiasthmatic activity.

**Methods:**

Studying the *in vitro* effect of fractions on isolated goat tracheal chain preparation and *in vivo* effect of fractions on milk induced eosinophilia, leukocytosis, clonidine-induced mast cell degranulation and capillary permeability in mice (n=5).

**Results:**

Methanol fraction of water extract of leaves of *Clerodendron phlomidis* (MFCP) at a dose of 4 mg/ml (49.41±1.462) and 10 mg/ml (52.38±0.872) significantly inhibited (*P*<0.05) the histamine (1.6 μg/ml) induced contractions in isolated goat tracheal chain preparation. In comparison to benzene, ethyl acetate fraction only methanol fraction at a maximum dose of 100 mg/kg i.p antagonized the eosinophilia by 62.32% and leukocytosis by 70.57% as compared to control group in mice (n=5) while significant percent protection offered to mast cells by 70.56% from degranulation as compared to control group. Also a significant reduction of capillary permeability 360±14.90 as compared to sensitized group 618.80±16.38 in mice was evident from its effect on optical density of the dye.

**Conclusion:**

The relative potency of methanol fraction of drug in various aspects of asthma confirms due to its antihistaminic, mast cell stabilizing, antiallergic and antistress action.

**121**

**The antianaphylactic effects of *Solanum xanthocarpum* flowers**

Alai VR^1^, Vadnere GP^1^, Patil MV^1^, Singhai AK^2^, Gaud RS^3^

**^1^Smt.S.S.Patil College of Pharmacy, Chopda, India; ^2^University of Sagar, Sagar, India; ^3^NIIMS, Mumbai, India.**

**Introduction:**

Potentiality of Kantkari flowers crude ethanol (95%) extract in various aspects of asthma has been prompted us to further to fractionate the polar constituents present in ethanol extract using benzene, ethyl acetate and methanol. All fractions of bioactive crude extract screened for its ability to alter the vascular permeability *in vivo* and histamine induced contraction *in vitro*.

**Methods:**

Studying the effect on isolated goat tracheal chain preparation and on Capillary permeability in mice (n=5).

**Results:**

Methanol fraction of ethanol extract of *Solanum xanthocarpum* flowers (MFSXEE) able to show a clear rightward shift in histamine–response curve in dose dependent manner than benzene and ethyl acetate fraction. Drug shown to have significant antagonistic effect (*P*<0.05) on histamine induced (1.6μg/ml) contraction at a dose of 2, 4, 10 mg/ ml (70.12±1.269, 56.09±1.928 and 48.17±0.787 respectively). This indicates the relaxant effect exerted by depression of histamine H1 receptor may be due to presence of flavonoids, phytosterols or saponins in polar methanol fraction. As well treatment group with benzene, ethyl acetate and methanol fractions at a dose of (25, 50, 100 mg/kg i.p) shows value of significance (*P*<0.05) with methanol fraction (MFSXEE) at highest dose of 100 mg/ kg i.p. (313.25±15.80) as compared to control group (618.80±16.38) which was sensitized and challenged with bovine albumin and Freund’s adjuvant 0.05 ml /kg i.p. in mice.

**Conclusion:**

The results obtained indicate that the fraction possesses antihistaminic and antianaphylactic properties, which may account for its use as an antiasthmatic in traditional medicine.

**122**

**Antistress and antiallergic effect of *Cassia occidentalis* leaf in asthma**

Gurav HB^1^, Vadnere GP^1^, Patil TP^1^, Singhai AK^2^, Gaud RS^3^

**^1^Smt.S.S.Patil College of Pharmacy, Chopda, India; ^2^University of Sagar, Sagar, India; ^3^NIIMS, Mumbai, India.**

**Introduction:**

Crude extracts of leaf of *Cassia occidentalis* under investigation were screened for their traditional anti-stress and antiallergic ability as excessive stress i.e. increases in TLC (Total leukocyte count) or nervous debility and blood eosinophilia / leukocytosis may aggravates the symptoms of asthma. Diagnostic evaluation and precise practical clinical approach to study the effect of crude extracts on milk-induced leukocytosis / eosinophilia in mice was used as after parenteral administration of milk there was increase in TLC and eosinophil count and this stressfull, allergic condition can be made normalized by administration of an antistress or adaptogenic and antiallergic drug.

**Methods:**

Studying the *in vivo* effect of crude extracts on milk induced eosinophilia and leukocytosis, in mice (n=5).

**Results:**

Ability of petroleum ether, ethanol and water extract of leaves of *Cassia occidentalis* (COPEE, COEE and COWE) was tested to control the milk induced eosinophilla at varying doses of 25, 50, and 100mg/kg i.p. Treatment group of COEE at a dose of (100 mg/ kg, i.p.) significantly (*P*<0.05) reduced milk induced eosinophilia (19.6±1.077) compared to other extracts and control. COEE potentiality in antagonizing the milk induced blood eosinophilia was found to be 54.35% while ethanol extract significantly (*P*<0.05) at a dose of 50 and 100mg/ kg i.p was able show notable inhibition of leukocytosis 70.36% and 77.57% respectively.

**Conclusion:**

Value of statistical significance supports the utility of present polar constituents of *Cassia occidentalis* as antistress and antiallergic agent in asthma.

**123**

**Effect of *Sphaernthus indicus linn* on gentamicin induced acute renal failure in rats**

Srinivasan KK, Jessy EM, Anand VM, Alex J

**Manipal College of Pharmaceutical sciences, Manipal University, Manipal, India.**

*Sphaeranthus indicus* (Asteraceae) is a medicinally important plant used as folk medicine. Several populations in northern India use this plant to control diabetes. The plant is also used for the treatment of urethral ailments, chronic skin diseases and jaundice. As very significant antioxidant activity has been observed in our laboratory with this plant, it was thought worthwhile investigating the nephroprotective activity of *S.indicus*. There is no report in literature of any earlier work on this activity. The ethanol extract of the entire plant material was subjected to nephroprotective screening in gentamicin induced acute renal injury in albino male wistar rats. Gentamicin induced renal injury resulted in elevated biochemical markers namely blood urea and serum creatinine followed by decreased total protein, serum albumin. The histopathological feature was acute tubular necrosis. The ethanolic extract of *S. indicus* at the dose level 300 mg/kg was found to normalize the changed blood urea, serum creatinine, total protein and serum albumin and bring about near to normal recovery in the kidneys, as evidenced microscopically. While investigating into the possible protective effect of *Sphaeranthus indicus*, it was observed that 10 days administration of the said extract in the prophylactic regimen prior to gentamicin administration (80 mg/kg, single dose, i.p), prevented the renal injury as indicated by normalized biochemical parameters and histopathological features. The findings suggest that the ethanol extract of *S. indicus* possesses nephroprotective activity with minimal toxicity and could offer a promising role in the treatment of acute renal injury inflicted by adverse effects of drugs like gentamicin.

**124**

**Biochemical studies on the anti-*ulcerogenic* potential of *Rubus llipticus***

Sai Nadh AVSR, Vadivelan R, Shanish A, Satish Kumar MN, Elango K, B Suresh

**JSS College of pharmacy, Ooty, India.**

*Rubus ellipticus* belongs to Rosaceae family is one of the 57 species occurring in India, out of total 400 species of genus *Rubus*. Traditionally is used for gastralgia, wound healing, dysentery, antifertility and many more. The presence of phytochemicals such as tannins, glycosides, triterpenes and saponins in the alcoholic extract was revealed by the preliminary phytochemical screening, some triterpenes compounds such as nimbidin have been shown to possess anti-ulcer activity. Keeping in view of medicinal importance the present study is an effort to investigate the anti-ulcer property of the ethanolic root extract of *Rubus ellipticus*. Antiulcerogenic effect of ethanolic root extract was studied in HCl/ethanol and aspirin plus pylorus ligation model. During the acute toxicity study the ethanolic extract at dose level of 2000mg/kg did not showed any sign of toxicity and mortality. Based on the acute toxicity studies three dose level were selected (125, 250 and 500mg/kg) and were grouped into five groups of 6 animals in each group and the animals were given doses of 125, 250, 500mg/kg of ethanolic extract, positive control(omeprazole 2mg/kg) and control (distilled water). The extract reduced ulcer area and ulcer index in HCl/ethanol model and reduced ulcerative lesions, free and total acidity, total proteins but raised total carbohydrates and the pH of gastric juice in aspirin plus pylorus ligation model and hence possess significant antiulcer activity

**125**

**Study on anti-stress activity of nut extract of *Semecarpus anacardium*, linn. in rats**

Ahmed Faiz, Verma Richa, Shivaraj Gouda T, Venkat Rao N, Shalam MD

**Vutkoor Laxmaiah College Of Pharmacy, Raichur, India.**

**Introduction:**

The objective of the present study is to evaluate anti-stress activity of milk extract of nuts of *S. anacardium* Linn, (Anacardiaceae). The extract was primarily subjected for preliminary phytochemical investigation and for acute oral toxicity studies (LD_50_).

**Methods:**

Anti-stress activity was evaluated in cold restrained stress in rats. Various biochemical parameters like Glucose, Cholesterol, Triglyceride, BUN, SGOT and SGPT and the wt. and vol. of organs like liver, spleen, kidney and adrenal glands were estimated.

**Results:**

The milk extract of nuts of *S. anacardium* was fond to contain flavonoids, carbohydrates and phenolic compounds. The extract was found safe upto the dose level of 2000mg/kg. Cold restrained stress model altered various biochemical parameters like GLU, CHOL, Triglyceride, BUN, SGOT and SGPT and the wt. and vol. of organs like liver, spleen, kidney and adrenal glands. The medium and high doses (200 mg/kg and 400 mg/kg) have reduced stress induced elevated levels of GLU, CHOL, Triglyceride, BUN, SGOT and SGPT, decreased the wt. and vol. of the liver, kidney, adrenal gland (4) an increased weight of the spleen.

**Conclusions:**

The present investigation revealed that milk extracts of nuts of *S.Anacardium* exhibited anti-stress activity by stabilizing bio-chemical parameters, wt. and vol. of the organs.

**126**

**Diuretic activity of seeds of *Cucurbita maxima* duchesne in albino wistar rats**

Jose MA, Balasubramanian S, Rahman AH, Varghese NA, Kumar AS, Sumaiya A, Vivekanandhan S, Sridharan C, Radhakrishnan M

**Swamy Vivekanandha College of Pharmacy, Thiruchengode, Tamil Nadu, India.**

**Introduction:**

The seeds of *Cucurbita maxima* Duchesne (Red gourd) are used traditionally as diuretics and other urinary diseases. In this study we have evaluated the aqueous extract for the diuretic activity in albino Wistar rats.

**Methods:**

Male Wistar rats weighing between 120 to 180g are divided into three groups of six animals each. Animals were deprived of food for 18h before the experiment. They were hydrated with 5ml/kg of water orally prior to the drug administration. Normal saline (1ml/100g) and Furosemide (10mg/kg) served as control and standard respectively. The drug (500mg/kg) was administered orally. Immediately after dosing the animals were placed in metabolic cages individually at room temperature. The urine was collected in measuring cylinder up to 18h after dosing. The concentration of Na+ and K+ in urine was also determined by flame photometer. The volume of urine and Na+ and K+ concentration of test group was compared with the control group.

**Results:**

The results revealed that the aqueous extract of seeds of *Cucurbita maxima* showed significant increase in urine volume (3.70 ± 0.09ml; *P*<0.01) when compared to control group. The activity was comparable with standard drug Furosemide (5.45 ± 0.48ml; *P*<0.01). But the excretion of Na+ and K+ in urine was not significantly increased in drug treated group when compared to control group.

**Conclusion:**

The aqueous extract of *Cucurbita maxima* showed significant diuretic activity. This study confirmed the traditional claims.

**127**

**Hepatoprotective activity of SR-101, a polyherbalformulation**

MD Shamsuddin Munawar, MD Ashwaq Hussain1, Venkat Roa N, Shivaraj Gouda T MD Shalam

**Vutkoor Laxmaiah College of Pharmacy, Raichur, India.**

**Introduction:**

To study the hepatoprotective activity of polyherbal formulation, SR-101

**Methods:**

The polyherbal formulation, SR- 101 consisting of medicinal plants *Phyllanthus niruri, Plumbago zeylanica, Bacopa monniera, Eclipta Alba, Picrorrhiza Kurrooa, Boerhaavia diffusa*, was brought from SHRUSHTI, an Herbal Pharma industry in Bangalore, and was subjected to acute toxicity studies as per OECD guidelines no. 425. The hepatoprotective activity was evaluated in rats by thioacetamide induced hepatotoxicity model, using various functional (thiopentone induced sleeping time), physical (wet liver weight and wet liver volume), biochemical parameters and histological features of the liver sections.

**Results:**

The polyherbal formulation, SR-101 was subjected for LD_50_ study and even up to dose level of 2000mg/kg it has not produced any lethal effect. The formulation, SR-101 was tested on thioacetamide (hepatotoxicant) induced hepatotoxicity model in rats. Toxicant group have shown an increased wet liver weight and wet liver volume which was markedly reduced in SR-101 treated groups. SR-101 reduced the elevated levels of ALT, AST, ALP, direct and total biliruibin, cholesterol and triglycerides. The reduced levels of total proteins and albumin were elevated in SR-101 treated groups when compared to toxicant group. SR-101 significantly reduced the thiopentone induced sleeping time as it was significantly elevated in thioacetamide treated groups. Histopathological changes i.e., fatty changes (steatosis), necrosis etc. due to above hepatotoxicant were partly prevented in animals treated with the SR-101.

**Conclusion:**

The present investigation revealed that the polyherbal formulation, SR-101 found to possess hepatoprotective activity.

**128**

**Pharmacological and bio-molecular evaluation of *Vitex negundo* for its anti-asthmatic activity in wistar rats**

Prasanna Maruthi, Elango K, Shanish Antony A, Ambhore N, Santhi Vardhan C, Vadivelan R, Satish Kumar MN, B Suresh

**JSS College of Pharmacy, Ooty, India.**

The most common respiratory disorders which have been studied are asthma and chronic obstructive pulmonary disorders. Conventional treatment uses drugs to either control symptoms or relieve the discomfort but can never eliminate the ailment. *Vitex negundo* has been ethnopharmacologically documented to be used in treating respiratory disorders. Ethanolic leaf extract of *Vitex negundo* (VNLE) and crude powder of *Vitex negundo* (VNCE) at a dose of 100mg/kg body weight were evaluated for antiasthmatic activity in ovalbumin induced asthma in wistar albino rats. The total WBC count present in bronchoalveolar lavage fluid (BALF) was higher in control animals when compared with VNLE and VNCP. Our results also suggest that the production of nitrate was significantly reduced by VNLE and VNCP. VNLE and VNCP significantly decreased (*P*<0.001) the serum IgG and tissue *malonyldialdehyde* (MDA), *myeloperoxidase* (MPO) levels and brought the values to normal. So by concluding all the above factors suggested that *Vitex negundo* leaf or aerial parts can be evaluated for treating clinical asthmatic disorder.

**129**

**Evaluation of hypocholesterolemic activity of ethanolic extract of *Gardenia gummifera* linn. gum resin in high cholesterol diet induced and triton WR-1339 induced hypercholesterolemia in rats**

Gajjar AV, Jaiswal SJ, Patel JA

**Institute of Pharmacy, Nirma University of Science and Technology, Ahmedabad, India.**

**Introduction:**

Traditionally *Gardenia gummifera* has been used as a folklore medicine in obesity and lipolytic disorders. The aim of the present study was to investigate the role of gum resin in reducing cholesterol levels in High Cholesterol Diet induced and Triton WR-1339 induced hypercholesterolemia in rats.

**Materials and Methods:**

The High cholesterol diet (HCD) model consisting of 1% cholesterol, 0.5% cholic acid, 5% lard oil was fed to the animals (40 days) and in Triton WR-1339 model, Triton WR-1339 was administered (300mg/ kg i.p. on 28^th^ day). In both the models, ethanolic extract of *Gardenia gummifera* was administered to the treatment groups orally at doses of 100, 200, 400 mg/kg in 0.5% CMC (40 days). Atorvastatin (2 mg/ kg in 5% CMC) was used as standard drug.

**Results:**

Administration of ethanolic extract of gum resin normalized the changes induced by high cholesterol and Triton on serum, hepatic and fecal parameters evaluated for hypercholesterolemia, oxidative stress and atherogenic risk predictors along with enhanced excretion of fecal cholesterol in dose dependent manner. The results were comparable with standard drug and were supported by restoration of normal architecture of liver and aorta as revealed from histological findings.

**Conclusions:**

Ethanolic extract of *Gardenia gummifera* gum resin possesses cholesterol suppressive capacity and antioxidant activity. It also has ability to attenuate the accelerated development of atherosclerosis in hypercholesterolemic rats.

**130**

**Study on hepatoprotective activity of leaf extract of *Cassia sophera* linn. in rats**

Suhael Ashraf, Bhosale Kiran H, Venkat Rao N, Shivaraj Gouda T, Shalam MD

**Vutkoor Laxmaiah College of pharmacy, Raichur, India.**

**Introduction:**

*Cassia sophera* linn (*Caesalpiniaceae*) is a plant with a variety of ethnic medicinal uses. Present study is planned to screen hepatoprotective activity of the aqueous extract of leaves of *Cassia sophera*.

**Methods:**

Powdered leaves were extracted with water and were subjected for phytochemical investigation and LD_50_ study was done as per OECD guidelines no. 425. Hepatoprotective activity of aqueous extract was studied against ranitidine induced hepatotoxicity in rats. Functional (thiopentone induced sleeping time), physical (wet liver weight and wet liver volume), biochemical and histopathological changes of livers, thipentone sleeping time were assessed.

**Results:**

Phytochemical investigations revealed the presence of carbohydrates, amino acids, fixed oils, fats, glycosides and sterols in aqueous extract of *Cassia sophera*. Extract has not produced any mortality up to the dose level of 2g/kg. In comparison with the ranitidine toxicant group the extract treated group reduced wet liver weight and wet liver volume. It also reduced the levels of SGPT, SGOT, SALP, cholesterol, triglycerides, direct and total bilirubin and thiopentone induced sleeping time. The reduced total proteins and albumin levels were elevated. The histopathological changes i.e. fatty changes (steatosis), necrosis etc were partly prevented in animals treated with the extract.

**Conclusion:**

It can be concluded that aqueous extract of leaves of *Cassia sophera* showed significant hepatoprotective effect against ranitidine induced hepatic damage as depicted by functional, physical, biochemical and histological changes in liver.

**131**

**Antioxidant and antitumor activity of alcoholic extract of stem bark of *Holoptelea integrifolia***

R Vijay^1^, Manjula SN^1^, Mruthunjaya K^2^, Vipan PK^1^, Gopalan KN^1^, Mallikarjuna RC^1^

**^1^Manipal College of Pharmaceutical Sciences, Manipal, India;**

**^2^JSS College of Pharmacy, Mysore, India.**

**Introduction:**

In Indian traditional medicine, the bark of *Holoptelea integrifolia* (family: Urticaceae) is used to treat intestinal tumors and paste of the bark is externally applied to treat the inflammation of lymph glands. Therefore the present study was undertaken to investigate the possible antitumor and antioxidant activity of *Holoptelia integrifolia* stem bark extract.

**Methods:**

*In vitro* antioxidant activity of alcoholic extract of *Holoptelea integrifolia* was tested in three well established methods. *In vitro* cytotoxicity in Ehrlich Ascites Carcinoma [EAC] and Dalton’s Ascites Lymphoma was assessed by Trypan blue exclusion method and MTT bioassay was used to determine *in vitro* cytotoxicity in human cancer lines [HeLa and MCF-7]. The EAC and DLA inoculated mice were used to assess cytotoxicity in liquid and solid tumor respectively. In EAC model, mean survival time [MST], hematological parameters, endogenous antioxidant enzymes were assessed. However, in solid tumor only tumor volume and weight were recorded.

**Results:**

Extract showed significant *in vitro* antioxidant activity in the tested methods. Extract also caused significant *in vitro* cytotoxicity in EAC, DLA, MCF-7 and HeLa cell lines. In liquid tumor, extract at 100mg/kg (i.p) was found most effective by increasing the MST, maintained normal hematological parameters and endogenous antioxidant enzymes. In solid tumor, significant reduction in tumor volume and weight was observed in animals treated with extract at the same dose.

**Conclusion:**

Extract showed significant *in vitro* antioxidant and cytotoxic activity. In *in vivo* study the extract at 100mg/kg was effective in reversing all the parameters in EAC and DLA inoculated mice.

**132**

**Kiwi extracts protect HepG2 cells from t-BHP induced oxidative damage**

Heekyoung Y, Hyun Ju H, Won young K, Chang Hoon H, Young Jae L

**Cheju National University, Jeju, Korea.**

In this study, we investigated the protective effect of 80% methanol extracts from various kiwi fruits on tert-butylhydroperoxide (t-BHP)-induced cytotoxicity in a human hepatoma cell line (HepG2). Kiwi extracts significantly reduced t-BHP-induced oxidative injury in HepG2 cells in a dose-dependent manner, based on the tests of cell cytotoxicity, lipid peroxidation, DNA damage, and reactive oxygen species (ROS) levels. In addition, the extracts reduced the expression of apoptosis-related proteins, such as cleaved caspase 3 and cleaved PARP protein. Moreover, kiwi extracts reduced the levels of 1, 1-diphenyl-2-picrylhydrazyl (DPPH) radicals, superoxide radicals and lipid peroxidation. The results suggest that kiwi extracts have potential protective effect against oxidative damage induced in HepG2 cells. The results could be beneficial for liver diseases where it is known that oxidative stress plays a crucial role.

**133**

**Anti-obesity effects of jeju dangyuja peel on high fat diet-induced obesity rats**

Hyun Ju H^1^, Heekyoung Y^1^, Won Young K^1^, Young Hun C^2^, Young Jae L^1^, Chang Hoon H^1^;

**^1^Cheju National University, Jeju, Korea; ^2^National jeju Agricultural Experiment station, R.D.A, Jeju, Korea.**

Jeju Dangyuja (*Citrus grandis Osbeck*) is one of the native citrus species which have been used as a folk remedy in Jeju island. In this study, we investigated the anti-obesity effects of Jeju Dangyuja peel on high fat diet-induced obesity rats. Male Sprague-Dawley (SD) rats were divided into 4 groups; (1) normal diet (ND)group, (2) high fat diet (HFD)group, (3) HFD with Dangyuja peel treated group, (4) HFD with sibutramin treated group. Each groups were treated for 10 weeks, and body weight, food intake, fat weight (epididymal, peritoneal), and serum lipids profiles (triglyceride, LDL-cholesterol and total cholesterol) were measured. Dangyuja peel treated group showed significantly reduced body weight, and reduced weight of epididymal and peritoneal fat compared with HFD group. The levels of triglyceride, LDL-cholesterol, and total cholesterol in blood serum were significantly decreased in Dangyuja peel treated group compared with those of HFD group. The fat liver formation induced by HFD was significantly reduced in rats administrated with Dangyuja peel. The results suggest that Dangyuja peel could prevent obesity and metabolic disease associated with hyperlipidemia.

**134**

**Wound healing activity of *Abroma augusta* in wistar rats**

Shenoy S^1^, Shanbhag T^1^, Dattachaudhuri A^2^, Bairy KL^1^

**^1^Kasturba Medical College, Karnataka, India; ^2^A.J.Shetty Medical College, Karnataka, India.**

**Introduction:**

*Abroma augusta* has been used traditionally for the treatment of sores. The wound healing profile of alcoholic extract of *Abroma augusta* and its effect on dexamethasone suppressed wound healing was evaluated in Wistar rats.

**Methods:**

An alcoholic extract of *Abroma augusta* was prepared. Three models were used - incision, excision and dead space wound models. Four groups of animals were used for each model. They were administered 2% gum acacia, *A.augusta*, dexamethasone and *A.augusta* with dexamethasone respectively. The parameters studied included breaking strength of incision wound, epithelization and wound contraction rate in the excision wound model, breaking strength, dry weight and hydroxyproline content of granulation tissue in dead space wound model.

**Results:**

The breaking strength of incision wound of *A.augusta* treated group was significantly increased (*P*<0.000) while that of dexamethasone treated animals was significantly decreased (*P*<0.000) as compared to control. Co-administration of dexamethasone and *A.augusta* significantly reversed the dexamethasone suppressed wound healing in incision wound model (*P*<0.001). Animals treated with dexamethasone and *A.augusta* showed significant reduction in the period of epithelization as compared to rats treated with dexamethasone alone (*P*<0.011). Co-administration of dexamethasone and *A.augusta* significantly (*P*<0.004) increased the breaking strength of granulation tissue in the dead space wound model as compared to dexamethasone treated group.

**Conclusion:**

The alcoholic extract of Abroma augusta was found to increase the wound breaking strength and reverse dexamethasone suppressed wound healing.

**135**

**Evaluation of analgesic activity of hydro alcoholic leaf extract of *Passiflora foetida***

Chan Basha S K, Bajrang Raj D, Shashidhar Swamy B, Jayaram Reddy M, Shivaraj Gouda T

**V l College of Pharmacy Raichur, India.**

**Objective:**

To evaluate the analgesic activity of hydro alcoholic leaf extract of *Passiflora foetida*.

**Methods:**

Eddys hot plate method, In this method heat is used as a source of pain in mice. The drug *Passiflora foetida* leaf was given in two different doses ie 200mg/ kg, 300mg/kg and aspirin is used as standard drug in the dose of 25mg/kg.Extracts were administerd to animals and the reaction of animals was noted on the hot plate at 15, 30, 60and 120 minutes after the drug administration. Calculate percent increase in reaction time at each time interval.

**Results:**

The analgesic effect of hydro alcoholic leaf extract of *Passiflora foietida* was found to be significant against pain induced by eddys hot plate. There was a significant increase in reaction time of mice.

**Conclusion:**

Results suggest that the two different doses of *Passiflora foetida* leaves 200mg/kg, 300mg/kg hydroalcoholic extract, possess significant analgesic activity

**136**

***In vitro* antimicrobial and antioxidant potency of leaves of few medicinal plants of Gujarat region**

Kaneria M, Vaghasiya Y, Parekh J, Chanda S

**Saurashtra University, Rajkot, Gujarat, India.**

Recently, multiple drug resistance has developed due to indiscriminate use of commercial antimicrobial drugs commonly used in the treatment of infectious disease. The aim of this work was to investigate the *in vitro* antimicrobial and antioxidant potential of few medicinal plants from Gujarat region. The leaves of three plants viz. *Diospyros ebenum* Koen. (Ebenaceae), *Guazuma ulmifolia* L. (Sterculiaceae) and *Manilkara zapota* L. (Sapotaceae) were evaluated for their antimicrobial activity, DPPH free radical scavenging activity, total phenol and flavonoid content using successive extraction by cold percolation method with different solvents (petroleum ether, ethyl acetate, methanol and water). *In vitro* antimicrobial activity was tested against five bacterial strains viz. *Bacillus subtilis* ATCC6633, *Staphylococcus aureus* ATCC29737, *Pseudomonas aeruginosa* ATCC27853, *Salmonella typhimurium* ATCC23564 and *Enterobacter aerogenes* ATCC13048 by agar well diffusion method. Preliminary qualitative phytochemical analysis was also done for various phytoconstituents. Among the three plants, the methanolic extract of *D. ebenum* showed good antibacterial activity, DPPH free radical scavenging activity, phenolic content and also flavonoid content. In all the three plants, cardiac glycosides, tannins and triterpenes were more as compared to other phytoconstituents. The results obtained suggest that *D. ebenum* can be used in treating diseases caused by the test organisms.

**137**

**In vitro and *in vivo* anti diabetic activity of various fractions of *Schrebera swietenioides* roxb**

T Anjali, Babu RK, Gopalan KN, Mallikarjuna RC

**Manipal College of Pharmaceutical Sciences, Manipal, Karnataka, India.**

**Introduction:**

In traditional medicine the root of *Schrebera swietenioides* Roxb. was used for diabetes in the form of paste. Therefore the present study was undertaken to explore possible antidiabetic activity.

**Methods:**

The roots of the plant were extracted with absolute alcohol by soxhlet extraction and fractionated into dichloromethane (DCM), ethyl acetate (EA) and butanol (BUT) fractions. Aqueous extract was prepared by maceration. Diabetes was induced in rats by low dose STZ following high- fat diet. The antidiabetic effect of extract was evaluated by parameters like blood glucose (FBS), body weight, lipid profile (serum cholesterol, serum triglycerides, HDL-C), protein catabolism (total protein, creatinine). In vitro anti diabetic activity was evaluated by inhibition of non enzymatic glycosylation of haemoglobin method and Advanced Glycation End products inhibition assay (AGE inhibition).

**Results:**

In inhibition of haemoglobin glycation assay, 1mg/ml concentration of aqueous (MAC), ethyl acetate (EA), butanol (BUT), dichloromethane (DCM) showed percentage inhibition as: 74.3%, 78.3%, 58%, 75% respectively. Ethyl acetate fraction was found potent for haemoglobin glycation inhibition. Aqueous and ethyl acetate fractions showed AGE inhibition properties. In vivo results suggested that both alcoholic and aqueous fractions reduced FBS (fasting blood sugar) in diabetic rats significantly (*P*< 0.05). Neither fraction had any lipid lowering properties.

**Conclusion:**

*In vitro* results suggest that extracts of *Schrebera swietenioides* Roxb. have good AGE inhibition and haemoglobin glycation inhibiton activity. *In vivo* results suggest that alcoholic and aqueous extracts have moderate antidiabetic activity. The plant did not exhibit any hypolipidaemic activity.

**138**

**Muscarinic receptor binding and nootropic studies on Anximin*®*, a polyherbal formulation**

Kumar N^1^, Mishra S^1^, Kumar V^1^, Khanna VK^2^

**^1^Pharmacology Research Laboratory, Department of Pharmaceutics, Institute of Technology, Banaras Hindu University, Varanasi-221 005, India; ^2^Developmental Toxicology Division, Indian Institute of Toxicology Research, Lucknow-226 001, India.**

**Introduction:**

Anximin is a polyherbal formulation and its scientific validation is lacking. The objective of present study was to evaluate the putative nootropic activity of Anximin through validated animal models of learning and memory viz. transfer latency (TL) on elevated plus maze, passive avoidance (PA) and scopolamine induced amnesia. To elucidate the mechanism of action, a receptor binding study was also performed using rat’s hippocampus.

**Methods:**

Wistar rats (200 ± 20 g) were used for these experiments. Anximin was administered at two dose levels (20 and 40 mg/kg, p.o.) once daily for 7 consecutive days as suspension in 0.3 % carboxy methyl cellulose. Piracetam (100 mg/kg, p.o.) was used as standard. Behavioural experiments were performed 1 hr after last drug administration on day 7.

**Results and Conclusion:**

Anximin (40 mg/kg) significantly (*P* < 0.05) shortened the TL on day 1, 2 and 9. Anximin (20 and 40 mg/kg) significantly (*P* < 0.05) attenuated the amnestic deficits induced by scopolamine. Similarly in PA, only the higher dose of Anximin (40 mg/kg) produced a significant (*P* < 0.05) increase in step through latencies; along with a significant (*P* < 0.05) reversal of scopolamine induced amnesia of PA at both the dose levels. Results of receptor binding study indicated a significant (*P* < 0.05 and *P* < 0.01 respectively) increase in 3[H] QNB binding to muscarinic receptors in hippocampus with both the doses of Anximin. These findings indicate a possible nootropic action of Anximin, attributed to its action on muscarinic receptors.

**139**

**Dose respose relationship of fruit extract of *Terminalia chebula* on metabolic components in a rat model of metabolic syndrome**

Singh Inderjeet, Shafiq Nusrat, Malhotra Samir, Pandhi P

**PGIMER, Chandigarh, India.**

**Background:**

There is documented evidence of use of *Terminalia chebula* for various ailments in the Ayurvedic literature. The extract has shown to possess glucose lowering activity and improve insulin sensitivity in animal models of type 2 diabetes mellitus. The present study was carried out to study the dose response relationship of this extract in a rat model of metabolic syndrome.

**Methods:**

Six groups of rats were fed high fructose diet (HFD) for a period of 20 days to induce metabolic syndrome. Three doses of fruit extract of *Terminalia chebula* 50, 100 and 150mg/kg were administered orally and pioglitazone 2.7mg/kg was used as positive control. Blood samples were collected at day 0, 20 and 40 from the tail vein. Systolic blood pressure (SBP) was measured using tail cuff method and oral glucose tolerance test (OGTT) was done on the day of blood collection.

**Results:**

Administration of HFD for twenty days significantly increased fasting blood glucose (FBG), SBP, and area under curve of OGTT. On the day forty the FBG in the 50, 100 and 150mg/kg group was 97.33±5.82 (NS), 86.83±5.08 (p=0.038) and 85.67±6.74 (p=0.15) respectively. There was no significant difference in the SBP in the three doses of the extract at day forty.

**Conclusions:**

these results show that the fruit extract of *Terminalia chebula* exerts significant and dose dependent glucose lowering effect in rat model of metabolic syndrome.

**140**

**Wound healing activity of extracts of plantain banana (*Musa sapientum* var. *Paradisiaca*) in rats**

Agarwal PK, Goel RK

**Institute of Medical Sciences, BHU, Varanasi, India.**

Plantain banana (*Musa sapientum* var. *paradisiaca*, MS) has been shown to possess peptic ulcer protective activity. The present work with MS was undertaken with the premise that the drug promoting ulcer healing could have effect on wound healing also. Wound healing activity of MS was studied in terms of (i) percent wound contraction, epithelization period and scar area; (ii) wound breaking strength and (iii) on granulation tissue antioxidant status (estimation of superoxide dismutase (SOD) and reduced glutathione (GSH), free radical (lipid peroxidation, an indicator of tissue damage) and connective tissue formation and maturation (hexuronic acid, hydroxyproline and hexosamine levels) in excision, incision and dead space wound models respectively using standard procedures. The rats were given oral graded doses (50, 100 and 200 mg/kg/day) of aqueous (MSW) and methanolic (MSE) extracts of MS for a period of 10 to 21 days depending upon the type of study. Both MSW and MSE (100 mg/kg) showed optimal effect on wound contraction and epithelization in excision wound when administered for 21 days. Both MSW/MSE (100 mg/kg for 10 days) increased wound breaking strength and levels of hydroxyproline, hexuronic acid, hexosamine, superoxide dismutase, reduced glutathione in the granulation tissue and decreased percentage of wound area, scar area and lipid peroxidation when compared with the control group. The result of the present study thus, does indicate that plantain banana favoured wound healing which could be due to its antioxidant effect and on various wound healing biochemical parameters promoting the process of early keratinisation and healing.

**141**

**Sesamol, a phenolic antioxidant, attenuates chronic fatigue syndrome in murine water immersion stress model**

Sachdeva AK, Sethi R, Kuhad A, Chopra K

**University Institute of Pharmaceutical Sciences, Panjab University, Chandigarh -160014, India.**

Chronic fatigue syndrome (CFS) is an illness characterized by persistent and relapsing fatigue, often accompanied by numerous symptoms involving various body systems. The etiology of CFS remains unclear, but a number of studies have shown that oxidative stress and inflammation may be involved in its pathogenesis. The present study was designed to evaluate the effect of sesamol, a natural phenolic compound, in a mouse model of immunologically induced fatigue, wherein purified lipopolysaccharide (LPS) as well as Brucella abortus (BA) antigen were used as immunogens. Animals were subjected to forced swimming test session of 6 minutes on 1st, 7th, 14th, 19th and 21^st^ day; a significant increase in immobility time on 19^th^ day represented the CFS in mice. Biochemical analysis revealed that the chronic swim test significantly increased lipid peroxidation levels and decreased glutathione levels in mouse whole-brain homogenate. Serum tumor necrosis factor-alpha (TNF-α) levels were also markedly increased with LPS or BA challenge. Concurrent treatment with sesamol oil resulted in the significant reduction in the immobility time and there was significant attenuation of oxidative stress as well as TNF-α level. These preliminary findings suggest the pivotal role of oxidative stress and inflammation in the pathophysiology of CFS and that sesamol could be used as potential agent in the management of CFS.

**142**

**Ameliorative effect of *Punica granatum* flower extract in dextran sodium sulfate induced ulcerative colitis in mice**

Kaur R, Singh K, Singh N, Jaggi AS

**Department of Pharmaceutical Sciences and Drug Research, Punjabi University, Patiala (Punjab), India.**

Traditionally, *Punica granatum* is used for the treatment of diarrhea, dysentery and ulcerations. Therefore, the present study was designed to investigate the ameliorative role of *Punica granatum* L. in dextran sodium sulfate (DSS) induced ulcerative colitis in mice. Ulcerative colitis was induced by administering 2% DSS in drinking water for 7 days. Methanolic extract of Punica granatum L. was administered (100 mg/kg and 200 mg/kg *p.o.*) daily for 7 days i.e., starting from the day of colitis induction. Sulphasalazine (100 mg/kg *p.o.*) was used as standard drug. The extent and severity of ulceration was analyzed macroscopically and histopathologically. Biochemical analyses of myeloperoxidase (MPO), reduced glutathione (GSH), and tissue lipid peroxides (TBARS) was done for exploring the possible mechanism of actions of *Punica granatum*. Administration of DSS resulted in induction of severe ulcerative colitis in mice in the form of bloody diarrhea, colonic ulcerations, severe intestinal inflammation and extensive mucosal damage as assessed macroscopically and histopathologically. Biochemically, there was significant rise in tissue MPO, TBARS and reduction in GSH levels. Treatment with *Punica granatum* L. (100 mg/kg and 200 mg/kg p.o) significantly attenuated DSS induced bloody diarrhea, colonic ulcerations, intestinal inflammation and mucosal damage and also normalized biochemical alterations comparable to that of standard drug sulphasalazine. It may be concluded that *Punica granatum* ameliorates dextran sodium sulfate induced colitis in mice possibly through its anti-inflammatory and antioxidant actions.

**143**

**Antidiabetic activity of saponins of *Momordica cymbalaria* in streptozotocin–nicotinamide induced niddm mice**

Shubhapriya K, Koneri R, Sarvaraidu CH

**Visveswarpura Institute of Pharmaceutical Sciences, Bangalore 560 070, India.**

**Objective:**

To evaluate antidiabetic activity of saponins of *Momordica cymbalaria* in Streptozotocin.Nicotinamide induced NIDDM Mice.

**Materials and Methods:**

NIDDM in BALB/c mice was induced by a single intraperitoneal injection of Streptozotocin (100 mg/ kg, i.p) 15 min after the intraperitoneal administration of Nicotinamide (240 mg/ kg, i.p). Hyperglycemia was confirmed by elevated blood glucose levels determined on day 7 after injection. Saponin mixture was obtained from ethanolic extract of *Momordica Cymbalaria*. Saponins of MC (SMC) 175mg/kg, p.o/30days and Metformin350mg/kg, p.o/30days were administered to the NIDDM diabetic mice. At the end of the treatment serum was analyzed for glucose, cholesterol, Triglyceride and Insulin; Pancreas was studied for histological changes.

**Result:**

Treatment with SMC (175mg/kg, p.o/30 days) and Metformin (350mg/kg, p.o/30days) to NIDDM diabetic mice produced a significant fall in blood glucose, cholesterol, Triglyceride and increase in serum insulin level. Pancreatic islets and beta cells showed an increase in number.

**Conclusion:**

Saponins of MC have significant NIDDM antidiabetic activity and the activity; may be due by increasing insulin secretion probably by regeneration of pancreatic beta cells.

**144**

**Anti-inflammatory and antioxidant activity of *Strychnos nux vomica* linn**

Chaurasia S

**College of Engineering and Technology, IILM Academy of Higher Learning, 17-18, Knowledge Park II, Greater Noida, G.B. Nagar, U.P. 201306, INDIA.**

*Strychnos nux-vomica* Linn. (Loganiaceae) is used traditionally for the treatment of rheumatism and other inflammatory conditions. Antilipid peroxidative property of *S. nuxvomica* has been reported. It also possesses significant metal chelation property. The present study was undertaken to screen the relationship between antiinflammatory and antioxidant activity of *S. nuxvomica* seed extract. The anti-inflammatory activity of *S nuxvomica* extract was studied on acute and chronic phases of inflammation using carrageenan induced paw edema and the cotton pellet granuloma test, respectively. The efforts has been made to explain the mechanism of action by studying antioxidant property on enzymic and nonenzymic models of lipid peroxidation accompanied by measuring reduced glutathione level under normal and toxic condition. In order to evaluate antihepatotoxic activity of *S. nuxvomica* extract, level of serum transaminases (SGOT and SGPT) was measured. Extract (50–200 mgkg^−1^ b.w., p.o.), when tested against carrageenan induced paw oedema and cotton pellet-induced granuloma, exhibited dose and time dependent significant inhibition on both the models of inflammation. Enzymic and nonenzymic models of lipid peroxidation were induced by Fe^3+^-ADP (1.6 mM-62 μM) and FeSO_4_ (0.5mM) respectively. The extract inhibited both the models of lipid peroxidation in a dose dependent manner. ED_50_ was found to be 149μg/ml and 85μg/ml on FeSO_4_ and Fe^3+^-ADP models respectively. It significantly inhibited aerobic and FeSO_4_ induced depletion of GSH in time and dose dependent manner. Oral treatment of drug up to 300 mgkg^−1^ b.w. for 30 days did not show any rise in serum transaminases. Results indicate that the ethanol extract of *Strychnos nux-vomica* possess potent anti-inflammatory and antioxidant property with no detectable adverse effect.

**145**

**Pharmacological validation of a marketed ayurvedic formulation for its anti-inflammatory activity**

Joseph Serene, Elango K, Golhani Dilip, Rajeswari Raja, Sathish Kumar MN, Vadivelan R, Gunjan, S Suganya, Shanish Antony A, B Suresh

**J.S.S College of Pharmacy, Ooty, India.**

Inflammation is one of the underlying mechanism in several chronic disease conditions like coronary artery disease, asthma, Alzheimer’s, arthritis and some cancers. In the present study the formulation chosen is a classical polyherbal one containing ingredients of plant and mineral origin. The study involved the pharmacognostical and pharmacological validation of the formulation. The total ash, acid insoluble and water soluble ash were determined to be 39.86%, 12.89%, 33.65%respectively. The pharmacological validation involved acute models like carragenan, 5-HT and histamine induced rat paw edema. In the carragenan induced rat paw edema model, the 125 mg/kg and 250 mg/kg showed significant activity (*P*<0.001) at the end of fifth and fourth hour respectively. In histamine induced edema, significant activity (*P*<0.001) was produced with 125 mg/kg at the third hour, with 250 mg/kg at the second hour. Similar, significant results were obtained with 5-HT induced edema. Prolonged effects of the formulation was studied using cotton pellet granuloma and Freund’s adjuvant induced arthritis model. The efficacy of the formulation in the cotton pellet granuloma model was comparable to that of the positive control (*P*<0.001). Significant effects was also produced in Freund’s adjuvant induced arthritic model and brought the arthritic condition to near normal. The formulation at 0.1, 0.5 and 5 mg/ml exhibited significant membrane-stabilizing activity (*P*< 0.05) and significant mast –cell stabilizing activity (*P*< 0.001) at 10 and 100 μg/ml. Thus, it can be concluded that this preparation showed significant anti-inflammatory activity.

**146**

**Pharmacological and biomolecular investigation of curcumin for its anti-asthmatic activity**

S Suganya, Shanish A, Saikat S, Satish Kumar M N, Elango K, Vadivelan R, Gunjan, J Serene, V Radhika, B Suresh

**J.S.S College of Pharmacy, Ooty, India.**

Curcumin has been ethnopharmacologically documented for its use in respiratory and inflammatory ailments, and asthma is fundamentally a respiratory disorder involving mediators of inflammation. The aim of the present study was to investigate the effect of curcumin on bio-molecular parameters involved in asthma in rats. Asthma was induced in rats by sensitization with ovalbumin (OVA) except the normal (i.e. saline treated) group. Except the normal and the sensitized control (i.e. only OVA treated) groups the remaining three groups were treated with standard drug, curcumin (100 mg/kg) and (200 mg/kg) respectively. Bronchoalveolar lavage fluid (BALF) and lung tissue homogenate were obtained following sacrifice after 60 days of treatment and nitrate, malonyldialdehyde (MDA), myeloperoxidase (MPO) levels, and percentage of intact and granulated mast cells were determined. On treatment with OVA; nitrate (303.66±1.87), MDA (39.25±0.95), MPO (22.6±0.68) levels were elevated, whereas percentage of intact mast cells (13.5±2.3) was decreased in comparison to normal levels. The curcumin- II (i.e. curcumin, 100 mg/kg treated) group reverted values of different parameters closer to normal (i.e. saline treated) group values as compared to the values of curcumin-I (i.e. curcumin, 200 mg/kg treated) group but the standard treatment was most significant in normalizing values. Curcumin modestly restored altered bio-molecular parameters in asthma. But, the exact molecular mechanism by which curcumin reduces the levels of malonyldialdehyde (MDA) and myeloperoxidase (MPO) is still not resolved. So, it needs further pharmacological investigation and clinical trial study of curcumin in order to cultivate a potential restorative agent for the treatment of clinical asthma.

**147**

**Pharmacological evaluation of curcumin for its nephroprotective activity in 5/6 nephrectomized rat model**

Ambhore N, Shanish Antony A, Prasanna Maruthi, Patil M, Vadivelan R, Satish Kumar MN, Elango K, B Suresh

**J.S.S College of Pharmacy, Ooty, India.**

The present study was to investigate the effect of curcumin in Wistar rats in 5/6 nephrectomized rat model. The curcumin was administered at 100 mg/kg and 200 mg/kg orally for 60 days. After 60 days parameters such as body weight, blood pressure, haemoglobin, cholesterol, triglycerides were estimated. The kidneys from all the groups were subjected to histopathological evaluation. Treatment with curcumin 200 mg/ kg showed significant reduction of body weight, cholesterol and triglycerides levels as compared to nephrectomized control. A significant decrease in blood pressure and increased haemoglobin were observed at 200mg/kg curcumin. Histological study of kidney tissue showed no congestion of interstitium, tubular capillaries and glomerular tuft of capillaries without focal haemorrhage. Curcumin ameliorated the parameters by normalizing the elevated levels in experimental rats. Thus the efficacy of curcumin of curing or alleviating CRF is a potential herbal, medicine.

**148**

**Gastroprotective activity of ayurvedic formulation (AYF-1) in aspirin induced acute gastric ulcer**

Gunjan, Elango K, Krishnaprasad D, Satish Kumar MN, Vadivelan R, Shanish Antony A, Deb Roy Partha, J Serene, S Suganya, B Suresh

**J.S.S College of Pharmacy, Ooty, India.**

Gastric ulcer is a serious gastrointestinal disease which occurs due to excessive acid secretion in the stomach. Natural drugs mostly augment the defensive factors and may be slow in activity but are reliable and safe. The present study was undertaken to study the antioxidant and cytoprotective activity of the Ayurvedic formulation (AYF-1) in aspirin induced gastric mucosal damage in rats. The ayurvedic formulation (AYF-1) was evaluated at two dose levels (350mg/kg and 700mg/kg)orally. The parameters observed include ulcer index, mucin content, non-sulphhydryl(NP-SH), catalase(CAT), lipid peroxidase(LPO) and superoxide dismutase(SOD). The results indicate that the formulation at both dose levels(350mg/kg and 700mg/kg) produced significant reduction in the ulcer index but at 350mg/kg dose level, it was significant than the positive control in decreasing ulcer index (*P*<0.05) and at 700mg/kg dose level was equipotent to positive control. There was significant increase in the mucin content (*P*<0.01) and at 700mg/kg dose level is equipotent to positive control. There was significant increasing non-protein sulfahydryl(NP-SH) (*P*<0.01) but at 700mg/kg dose level was found to be equipotent to positive control. The antioxidant effect of formulation at both dose levels was capable of producing significant decrease (*P*<0.01) in catalase (CAT) and superoxide dismutase(SOD) and a significant decrease in lipid peroxide (LPO). It may be said that the formulation may have increased mucin activity and antioxidant property which is direct indication of gastric mucosal protection. Thus it can be concluded that this formulation (AYF-1) has antisecretory activity as well as gastroprotective activity. This synergistic activity in the formulation may be due to presence of multiple herbs. Hence this formulation can be used as antiulcer agent.

**149**

***Spirulina (arthrospira)* decreases the valproic acidinduced teratogenicity in ICR mice**

Chamorro-Cevallos G, Escalona-Cardoso G, Paniagua-Castro N, Pérez-Pastén R

**Escuela Nacional de Ciencias Biológicas, Instituto Politécnico Nacional, México D.F., México.**

The widely popular antiepileptic drug valproic acid (VPA) is a potent induced of neural tube defects in human and mouse embryos. Accumulating evidence suggests that its teratogenicity is associated with the potential of causing embryonic generation of free radicals and increased oxidative stress. On the other hand, *Spirulina* (Sp), is a blue-green alga whose pharmacological properties have been attributed to a great extent to its antioxidant potential. Accordingly, the present study was performed to investigate the influence of *Sp* on the teratogenicity of VPA in ICR mice and the possible mechanism of action. VPA in the form of sodium valproate was administered intraperitoneally to mice on gestation day (GD) 8 at a dose of 600 mg/kg. *Sp* was given orally at 125, 250 and 500 mg/kg daily from GD0 through GD18 (at which time the animals were sacrificed). Upon analysis the most common finding in fetuses after VPA exposition was exencephaly. The incidence of this and other malformations was significantly decreased in mice receiving *Sp*. Moreover, compared to the animals treated orly with VPA, in *Sp* supplemented mice, embryonic lipid peroxidation proved to be decreased, while the levels of superoxide dismutase, catalase and glutation peroxidase were found to be increased. In conclusion, these results illustrate the protective action of *Sp* through its antioxidant activity in relation to VPA-induced teratogenicity.

**150**

**Proguanil metabolic ratio and *CYP2C19* genotype in a south Indian population**

Chakradhara Rao US, Shewade D G, Adithan C

**Jawaharlal Institute of Postgraduate Medical Education and Research, Pondicherry, India.**

**Aim:**

To investigate the relationship between proguanil metabolic ratio (proguanil/cycloguanil) and *CYP2C19* genotype in a South Indian population.

**Methods:**

Fifty eight South Indians (18-55 years, 42 males, 8 females) were genotyped for *CYP2C19* (*2 and *3 alleles) by PCR-RFLP method. The 1.8 kb 5’ flanking region of *CYP2C19* was also sequenced, and Subjects were phenotyped for proguanil oxidation by collecting 5ml of blood 4.5 h after taking 200mg of proguanil HCl. Proguanil (PG) and cycloguanil (CG) concentrations were measured by high performance liquid chromatography. Metabolic ratios (PG/CG) of different genotypes were analysed by Mann-Whitney U-test.

**Results:**

Of the subjects genotyped, 16 were *CYP2C19* *1/*1, 26 were *CYP2C19* *1/*2, 6 were *CYP2C19* *2/*2, and 2 were CYP2C19*1/*3. The mean PG/ CG ratio (4.726) was lower in the homozygous wild type (*1/*1) subjects compared with that in heterozygous individuals (*1/*2; mean PG/CG = 9.629; P= 0.031) and individuals with homozygous mutant alleles (*2/*2; mean PG/CG= 9.914; p=0.040). There is no significant difference seen in mean PG/CG ratio between homozygous mutant subjects and heterozygous subjects with *2 allele. The promoter region polymorphisms of *CYP2C19* shall have influenced the conversion of PG to CG by altering the enzyme transcription.

**Conclusion:**

CYP2C19 enzyme role in cycloguanil formation *in vivo* was confirmed by the *CYP2C19* gene-dose effect. However, there was substantial overlap of PG/CG ratio in subjects of different *CYP2C19* genotypes, suggesting the influence of other variations in the promoter region or contribution of other enzymes to the formation of cycloguanil.

**151**

***In vitro* and *in vivo* activity of *Ocimum sanctum* against experimental systemic candidiasis in normal and immunocompromised mice**

Das A, Satish Kumar MN, Gupta S, Elango K, Vadivelan R, Shanish Antony A, Sethi N, Rajanandh MG, K Jayasankar, B Suresh

**J.S.S College Of Pharmacy, Ooty, India.**

*Candidiasis* is a common cause of morbidity and mortality among immunocompromised patients. Earlier reports reveal the antifungal activity (*in vitro*) of *Ocimum sanctum*. The present investigations evaluated the anticandidal activity of *O.Sanctum*. Hydro alcoholic extract of *O.sanctum* (HEOS) was obtained by cold maceration. The reports of phytochemical investigations found 0.75% of phenolic compounds and 2.5% of flavonoids in HEOS. *In vitro* investigations of HEOS at the dose range from 1000 to 15.625μg/ml were found to be effective against various fungal strains. Experimental systemic candidiasis was induced in mice by administering through tail vein (0.1ml of 10^6^ cells/ml of culture suspension of *C.albicans*). The animals were immunocompromised with cyclophosphamide (i.p). Oral administration of HEOS 200 and 400mg/kg body weight for 16 consecutive days showed highest mortality (40%) in untreated mice compared with treated. On day 16 animals were sacrificed and colony forming units (CFU) in various organs were recorded. A highly significant (*P*<0.01) decrease in CFU was found in HEOS treated mice compared to untreated in both normal and immunocompromised conditions. Histopathology of various organs (liver, kidney, intestine and lungs) exhibited similar results. There was no change in body weights of drug treated mice and a significant increase in total leukocyte count and lymphocytes compared to immunocompromised mice. It indicates hydroalcoholic extract of *O.sanctum* possess significant anticandidal activity in both normal and immunocompromised mice at tested dose levels and it could be due to bioactive principles like flavonoids and phenolic compounds distributed in HEOS.

**152**

**Anticandidal activity of *Ocimum basilicum* in normal and immunocompromised mice under gastrointestinal colonization**

Sethi N, Satish Kumar MN, N Neema, Elango K, Vadivelan R, Shanish Antony A, Das A, Rajanandh MG, B Suresh

**JSS College of Pharmacy, Ooty, India.**

*Candidiasis* is often observed as causal agent of oral and vaginal infections in immunocompromised individuals. Sweet basil species belong to the *Ocimum* genus and *Lamiaceae* family has immunomodulatory, antimicrobial and antifungal activity. The present study investigated the anticandidal activity of *Ocimum basilicum* in normal and immunocompromised mice under gut colonization. Hydroalcoholic extract of *Ocimum basilicum* (HEOB) was obtained by cold maceration. The quantitative analysis report of HPLC study indicated presence of quercetin (6.22μg/ mg), rosamarnic acid (1.2gμ4/mg), rutin (0.88μg/mg) and eugenol (0.04μg/mg). Experimental systemic candidiasis under gut colonization was induced by administering clindamicin and gentamicin to all the groups for whole duration of experiment and infected by *C.albicans* (i.v) on the eleventh day. The animals were immunocompromised by i.p injection of cyclophosphamide on day 13 (300mg/kg/bwt) and 15 (150mg/kg/bwt). 200 and 400 mg/kg/ bwt of HEOB administered orally once daily for 24 consecutive days and mortality was observed. High percentage of mortality (40%) was recorded in non treated groups compared with treated. Maximum colonization evidenced in fecal samples and in various organs (liver, kidney, intestine and lungs) of untreated mice. However, a significant (*P*<0.001) reduction of colony forming units in fecal samples and organs were recorded in HEOB treated mice. Histopathology of various organs exhibited the similar findings. This study concludes that O.basilicum possess significant anticandidal activity in both normal and immunocompromised mice under gut colonization at tested dose levels and it could be due to its bioactive principles like quercetin, rutin, rosmarnic acid and eugenol distributed in HOEB.

**153**

**Phytochemical and toxicological investigations of *Zanthoxylum rhetsa* fruits**

Nayak Yogendra, Zanwar Sachin B, Gajera Falguni P, Shreedhara CS, Jayashree BS, Unnikrishnan MK

**Manipal College of Pharmaceutical Sciences, Manipal University, Karnataka-576 104, India.**

*Zanthoxylum rhetsa* (Roxb.) DC, (Family: Rutaceae) is a plant growing in Coastal Karnataka and Kerala. The fruits has strong aroma and the dried fruit rind is used as spice. Kirtikar and Basu, Nadkarni, and Dymock mentions the fruit has stimulant, astringent, aromatic, and digestive properties and is prescribed in urinary disease and dyspepsia. For rheumatism the fruit is given with honey. Because of the plant is under threat of extinction the medicinal and health benefits of this plant fruits were not been explored. Fruits collected from the local place, dried extracted in Soxhlet apparatus with 70 percentage V/V alcohol for complete extraction. The alcoholic extract is then concentrated using rotovapour. It was further fractionated with chloroform, petroleum ether, ethyl acetate and n-butanol. In the preliminary phytochemical studies we have found that the extract contains alkaloids, glycosides and flavonoids. It was found that all fraction of the alcoholic extract contains alkaloids. The Petroleum ether and chloroform fraction showed the test for phytosterols. The n-butanol fraction seems to be rich in saponins and flavonoids. All fractions are then studies for the *in vitro* antioxidant activity. It showed radical scavenging and inhibiting lipid peroxidation. Acute oral toxicity studies were carried out for the extracts in female rats as OECD-420 and it was found to be non-lethal up to 2g/ kg. Further toxicological and pharmacological screening is under progress.

**154**

**Evaluation of the antidiabetic activitiy of a polyherbal formulation in rats**

Hariprasad MG, Razdan R

**Al-Ameen College of Pharmacy, Bangalore, India.**

**Background and Objectives:**

Diabetes mellitus is a major health threat today. DIASANSAR is a polyherbal formulation containing plant drugs of known antidiabetic properties. In the present study, DIASANSAR was evaluated for its protective effect against insulin dependent diabetes mellitus in male Wistar rats.

**Methods:**

Insulin dependent diabetes mellitus was induced by administering streptozotocin 65 mg/kg i.p in Wistar rats. The rats showing an increase in the glucose levels >245mg/dl were selected for the study. The DIASANSAR was administered for 20 days. The degree of protection was determined by measuring the levels of serum glucose, cholesterol, triglycerides, creatinine and liver glycogen.

**Results:**

In normal rats which were subjected to the oral glucose tolerance test by administering a lower dose (450 mg/kg, p.o) and higher dose (900mg/kg, p.o) of Diasansar powder along with glibenclamide (500mcg/kg, p.o) followed by a glucose load of 10g /kg, p.o DIASANSAR powder exhibited antihyperglycemic activity by significantly reducing the serum glucose levels. The diabetic complications were evidenced by the increased levels of serum glucose, cholesterol, triglycerides and creatinine whereas decreased levels of serum HDL and liver glycogen. DIASANSAR powder (450 mg/kg, p.o) exhibited antihyperglycemic, antihyperlipidemic activity by significantly reducing the serum glucose, cholesterol, triglyceride levels. DIASANSAR powder (450 mg/kg, p.o) also exhibited decreased diabetes associated nephropathy evidenced by significantly reduced serum creatinine levels.

**Interpretation and Conclusion:**

The results indicate that the DIASANSAR showed dose dependent antidiabetic activity.

**155**

**Anti-cataract activity of *Apium graveolens* in alloxan induced diabetic rats**

Kamalesh DR, T Prakash, Goli Divakar, Swamidasan R, Dixit P

**Acharya and B, M Reddy College of Pharmacy, Bangalore-560 090, Karnataka, India.**

**Objective:**

Diabetes the major metabolic disorder plays an major role in the pathogenesis of chronic complications such as retinopathy and cataract by increasing the amount of advanced glycation end products in tissues which alters the protein structure and function. Serendipity played an important role in many medical advances and also opens the path to discover remedies from natural resources. Past from many years plants and their extracts have been used as folklore medicine. Hence the present study was attempted to screen anti-cataract activity of *Apium graveolens*.

**Method:**

The present project was assessed for ocular changes over a period of six weeks post alloxan administration in rats. Cataract was induced experimentally by administration of Alloxan monohydrate (single dose, 80mg/kg i.p). Suspension of the extracts was prepared by using 0.5% sodium CMC, rats are divided in to nine groups, like control, diabetic control, and three does of aqueous and methanolic extract groups (100, 250 and 500 mg/kg p.o). The anti-cataract activity was assessed by determining its effect on serum enzymes (ALT, AST), lens opacity, GSH, Superoxide dismutase, Catalase, Protein carbonyl in lens and eye lens protein analysis by SDSpage method, in control, diabetic control, standard and extracts treated groups.

**Result:**

Aqueous and methanolic extracts at 250 and 500 mg/kg showed significant decrease in food intake and also normalized the activities of serum enzymes, GSH, Superoxide dismutase, Catalase and also reduced the cataract index when compared with the diabetic control.

**Conclusion:**

The present investigation indicates that the aqueous and methanolic extract possesses anti-cataract activity.

**156**

**Evaluation of *in vitro* and *in vivo* antitumour activity of alcoholic and ethyl acetate extracts of premna herbacea roxb against tumor models in mice**

Dhamija Isha, Pai KSR, Setty MM, Manjula SN

**Manipal College of Pharmaceutical Sciences, Manipal University, Manipal-576104, Karnataka, India.**

Root nodules of Premna herbacea Roxb. (Family:-Verbenaceae) are claimed to be useful in ayurvedic system of medicine for the treatment of cancer. Root nodules of Premna herbacea Roxb, have not been worked for possible anticancer activity. Thus, the present study has been under taken to study the antitumour activity of alcoholic and ethyl acetate extracts of title plant in Dalton’s lymphoma ascites model and in vitro cyctotoxic activity in MCF-7 cells (Human Breast adenocarcinoma cells) by MTT assay. *In vivo* test of the extracts were performed by inducing solid tumor (DLA) in Swiss albino mice by injecting 10^6^ cell/ml of tumor cell suspension s.c. Anticancer activity of these extracts (250 and 500 mg/kg i.p) was compared with that of Cisplatin (3.5 mg/kg i.p) on parameters such as tumor volume and tumor weight. Both alcoholic and ethyl acetate extracts were found to cause significant cytotoxicity in MCF-7 cells by MTT assay. In solid tumor (DLA), the tumor weight in all treated groups at the end of fourth week was significantly lower (*P*<0.05) than control group. The tumor volume in treated group at the end of fourth week was significantly lower (*P*<0.05) than control group. The standard drug cisplatin, was able to reduce solid tumor weight and tumor volume.

**157**

**Evaluation of antidepressant and anxiolytic activity of *Cassine albens* (R.) Kosterm (celastraceae) in rodents**

Gagarani MB^1^, Patil PH^2^, Surana SJ^2^, Fursule RA^1^

**^1^H. R. Patel Women’s College of Pharmacy, Shirpur, India; ^2^R.C. Patel Institute of Pharmaceutical Education and Research, Shirpur, India.**

**Introduction:**

*Cassine albens* belonging to the category Celastraceae was used as traditional medicine by tribal people of toranmal region of North Maharashtra. The plant was famously known among these people as “Bhutakes”. This plant was suggested to be useful in the ailments such as hysteria, syncope, normal headache, in snake byte etc. It was reported that this plant may useful for psychosomatic disorders hence their need to evaluate this plant for antidepressant and anxiolytic activity.

**Methods:**

Preparation of Extract: Hydro alcoholic extract of stem bark of *Cassine albens* was prepared by extracting the coarse powder of bark in 50% methanol and equal amount of water by cold maceration process. The extracted liquid was concentrated and dry extract was utilized for evaluation of biological activity.

**Biological activity:**

The extract was screen for the target activity

- Neuropharmacological profile in mice
- Spontaneous locomotor activity
- Forced Swim Test in rat
- Tail Suspension Test in mice
- Elevated Plus Maze Test in rat.
- Marble Burying Behavior in mice

**Result and Conclusion:**

Hydro alcoholic extract of present plant was found to exert antidepressant and anxiolytic activity in rodent experimental model. Statistical calculation reveals that all the result was significant as compare to control group. We proposed that although the hydro alcoholic extract exerts an antidepressant and anxiolytic activity, further investigation require confirming the mechanism and the site of action of isolated compound from this plant.

**158**

**Evaluation of wound healing and anti-inflammatory activity using flowers of *Tabernaemontana divaricata* and *Tabernaemontana erecta***

Basavaraj HC^1^, K Deepthi^2^, Manjunath PM^1^, L Prakash^1^, G Divakar^1^

**^1^Acharya and BM Reddy College of Pharmacy, Bangalore, India; ^2^Acharya Institute of Technology, Bangalore, India.**

**Objective:**

The present study was undertaken to evaluate wound healing and anti-inflammatory activity using flowers of *T.divaricata* and *T.erecta.*

**Methods:**

Wound healing activity was carried by using excision wound model by using simple ointment I.P of T.divaricata and T.erecta (5% and 10% w/w) using Farmycetin (1% w/w) as standard. A wound of 500 sq.mm was made and homeostasis was maintained. The physical attributes of wound healing viz wound closure, epithelization and scar features were recorded on 4^th^, 8^th^, 12^th^, 16^th^ day. Anti-inflammatory activity was carried with ethanolic extract using carrageenan induced paw oedema method. Acute toxicity study was carried using OECD guidelines (250 and 500 mg/Kg p.o dose was fixed) and diclofenac sodium 25 mg/Kg was used as reference standard. The paw thickness was measured using plethysmometer and vernier callipers and the percentage inhibition of inflammation was measured.

**Results:**

*T.divaricata* and *T.erecta* ointment showed significant (*P*<0.001) decrease in wound area on 8^th^ day and complete wound healing was found on 16^th^ day when compared to standard drug. For anti-inflammatory activity carrageenan induced paw oedema was significantly reduced at 60 and 240 min gradually when compared to standard drug.

**Conclusion:**

The results of this study have shown that the ethanolic flower extract of *T.divaricata* and *T.erecta* possess wound healing ad anti-inflammatory activity. This justifies the traditional usage of this plant as healing and inflammatory remedy.

**159**

**Study of *Tephrosia purpurea* roots in bronchial asthma**

Deshpande S, Dave P, Shah G

**K B Institute of Pharmaceutical Education and Research, Gandhinagar-382023, India.**

The study was designed to investigate the effect of *Tephrosia purpurea* roots against experimental bronchial asthma models. The ethyl acetate extract was studied in experimental models of asthma like: Mast Cell degranulation by compound 48/80, Histamine induced bronchospasm, Passive cutaneous anaphylaxis and Bronchoalveolar lavage study. Dexamethasone (5mg/kg) was used as a reference standard. A significant protection of rat mesenteric mast cells from disruption caused by compound 48/80 was offered by Ethyl acetate extract (100, 250 and 500 μg/ml). The extract also offered significant protection against mast cell disruption caused by antigen. In the passive cutaneous anaphylaxis model, pretreatment with ethyl acetate extract significantly reduced the amount of dye leakage, compared to control, at the site of heterogenous antibody injection. Ethyl acetate extract at the doses of 250 mg/kg and 500 mg/kg significantly delayed the onset of convulsions in guinea pigs due to acute bronchospasm induced by histamine aerosols. As compared with unsensitized animals, significant increase in eosinophil and basophil count was observed in untreated- sensitized animals challenged with egg albumin. Ethyl acetate extract (500 mg/ kg) did not produce any significant difference in the count of all the types of WBC detected in the bronchial fluid of sensitized animals compared with untreated sensitized animals. It can be concluded from the study that Ethyl acetate Extract of *Tephrosia purpurea* roots possesses significant protection against bronchoconstriction and anaphylaxis. Stabilization of mast cells and inhibition of immediate hypersensitivity reactions appear to be involved in its mode of action.

**160**

**Effect of *Ficus racemosa* stem bark extract in type 2 diabetes induced by high-fat diet and low dose stz in rats: A mechanistic approach**

Ramya E, Veeresh PV, Prabhakar KR, Unnikrishnan MK

**Manipal College of Pharmaceutical Sciences, Manipal – 576 104. Karnataka.**

**Introduction:**

The bark, root and fruits of *Ficus racemosa* have been claimed to possess antidiabetic activity in traditional ayurvedic system. Thus, the present investigation was carried out to explore possible antidiabetic activity.

**Methods:**

Powdered bark of *Ficus racemosa* was extracted with 95% ethanol (Soxhlet) to obtain FRE (ethanolic extract) followed by water extraction to obtain FRW. Type 2 diabetes like condition was induced by a combination of high - fat diet (2 weeks) and single dose of streptozotocin (25mg/kg, i.p) in male wistar rats. Rats with fasting blood glucose levels >200mg/dl after 7 days of STZ were randomized into groups and treated with FRE in graded doses for 15 days. Pioglitazone was used as positive control. After completion of the study, blood glucose, oral glucose tolerance, lipid profiles, serum insulin, enzymatic and non enzymatic liver antioxidant levels were estimated.

**Results:**

Treatment with FRE (200 and 400mg/kg) reduced blood glucose, serum insulin, lipid levels and Homeostasis Model Assessment (HOMA) levels and improved glucose tolerance, suggesting improvement in fat fed/ STZ induced insulin resistance. FRE supplementation decreased oxidative stress by improving endogenous antioxidants. FRE inhibited PTP- 1B with IC_50_ 12.1mcg/ml, alpha-amylase with IC_50_ 1.87mcg/ml and partially inhibited alpha-glucosidase. FRE, at 200mcg/ml showed 42.5% inhibition of DPP IV and at 10mcg/ml showed 82.6% binding to PPAR-gamma. FRE exhibited concentration dependent stimulation of glucose uptake by skeletal muscles (hemidiaphragm).

**Conclusion:**

Results suggest that ethanolic extract of *Ficus racemosa* inhibits fat fed/ STZ induced insulin resistance, lipid abnormalities and oxidative stress.

**161**

**Effect of ethanolic *Embelia ribes* burm extract on methionine – induced hyperhomocysteinemia and oxidative stress in rat brain**

Ansari MN^1, 2^, Bhandari U^1^, Islam F^3^, Tripathi CD^4^

**^1^Faculty of Pharmacy, Hamdard University, New Delhi, ^2^Azad Institute of Pharmacy and Research, Lucknow, ^3^Faculty of Science, Hamdard University, New Delhi, ^4^Department of Pharmacology, Vardhman Mahavir Medical College, Safdarjung Hospital, New Delhi, India.**

The present study was designed to evaluate the effect of ethanolic extract of *Embelia ribes* Burm on methionine-induced hyperhomocysteinemia and oxidative stress in rats. Methionine (1 g/kg, per day for 30 days) administration to vehicle control rats produced a significant (*P*<0.01) increase in homocysteine and lactate dehydrogenase (LDH) levels in serum and lipid peroxides (LPO) levels in brain homogenates with concomitant decrease in glutathione (GSH) levels in brain homogenates, as compared to vehicle control rats. Administration of ethanolic *Embelia ribes* extract (100 and 200 mg/Kg per day for 30 days) to methionine treated hyperhomocysteinemic rats produced significant (*P*<0.01) decrease in homocysteine and LDH levels in serum. In addition, a significant (*P*<0.01) decrease in LPO levels and increase in GSH levels was observed in brain homogenates. The results were comparable to that of folic acid, a standard antihyperhomocysteinemic drug. The results of our study, for the first time, provide clear evidence that ethanolic *Embelia ribes* extract treatment possesses antihyperhomocysteinemic and antioxidant effect against methionine-induced hyperhomocysteinemia and oxidative stress in rat brain.

**162**

**Pharmacological evaluation of *holarrhena antidysenterica* (wall) for hypoglycemic activity in stz-induced diabetic rats**

Srinivas V, Supriya Mana, MD Asif Ansari, Basavaraj HC, Goli Divakar

**Acharya and BM Reddy College of Pharmacy, Bangalore, India.**

**Objective:**

Diabetes is one of the common metabolic disorders affecting worldwide population of the world. Insulin and oral hypoglycemic agents are the major players in the management of the diabetes. The ‘Bhavaprakasha’ has recommended the use of the fresh bark and seeds of *Holarrhena antidysenterica* in the treatment of diabetes. However, scientific data on antidiabetic activity of *Holarrhena antidysenterica* is not available. So, the present study is made to explore the anti-diabetic effect of methanolic seed extract of *Holarrhena antidysenterica* (MEHAD) in streptozotocin (STZ) induced diabetic rats.

**Methods:**

Diabetes was induced by single i.v. injection of STZ at the dose of 35 mg/kg and rats with FBS above 200 mg/dl were included in the study and randomly divided into four groups of ten animals (male) along with the control group. The diabetic rats were treated with glibenclamide (standard), MEHAD was administred for 18 days. The effect of MEHAD on body weight, FBS, serum cholesterol and triglycerides, total protein, blood urea, urine glucose and liver glycogen levels are estimated.

**Result:**

The diabetic rats treated with MEHAD showed significant reduction in fasting serum glucose, cholesterol, triglyceride, total protein, blood urea, urine glucose and protection from the loss of body weight and increase in liver glycogen content during the treatment, these effects were comparable with standard. This suggests that the MEHAD possess anti-diabetic activity.

**Conclusion:**

Current study shows that MEHAD has favourable effect on blood glucose levels, liver glycogen, serum lipids and body weight and useful as antidiabetic agent.

**163**

***In vitro* antioxidant activity of different fraction of leaves of *Polycarpaea corymbosa* linn**

Mruthunjaya K^1^, Manjula SN^2^, Hukkeri VI^3^

**^1^J.S.S.College of Pharmacy, Mysore, India; ^2^Manipal College of Pharmaceutical Sciences, Manipal, India; ^3^Indira Institute of Pharmacy, Sadavali (Devrukh), India.**

**Introduction:**

The leaves of *Polycorpaea corymbosa* (Family: Caryophyllaceae) are used in Indian traditional medicine for treatment of jaundice. Preliminary phytochemical screening revealed the presence of phenolics in the plant. This inspired us to study its antioxidant properties.

**Methods:**

Aqueous alcoholic (70%) extract obtained by cold maceration of leaves was fractionated into petroleum ether (PCPE), butanol (PCB), ethyl acetate (PCEA) and water (PCWF) fractions. The antioxidant potency of different fractions were evaluated using well established *in vitro* methods, such as DPPH radical scavenging, Beta Carotene Linoleic Acid Module System (B-CLAMS), Hydroxyl radical (·OH) scavenging and anti-lipid peroxidation. Also Total Phenolic Content (TPC) of different fractions was determined.

**Results:**

Among the different fractions PEA showed highly potent antioxidant activity which is comparable to that of standards. Different standards like Ascorbic acid (ASC), BHT, Mannitol and Silymarin were used in different methods. IC_50_ values of PEA and standards were found to be 1.38±0.62 and 2.00±0.35 microgram/ml in DPPH radical scavenging activity, 32.5±3.42 and 92.5±4.67 microgram/ml in B-CLAMS method, 5.95±1.39 and 3.25±0.46 microgram/ml in.  OH radical scavenging activity and 13.0±1.28 and 5.9±0.16 microgram/ml in anti-lipid peroxidation assay. Further TPC of PCPE, PCB, PCEA and PCWF were found equivalent to 27.56±3.41, 93.58±2.79, 216.01±5.59 and 32.59±2.79 mg of gallic acid respectively.

**Conclusion:**

The fraction containing high TPC (PCEA) showed highest antioxidant activity and the fraction containing least TPC (PCWF) showed least antioxidant activity. So it was concluded that total phenolics are responsible for antioxidant activity and ethyl acetate fraction was found to be a potent antioxidant.

**164**

**Anti-inflammatory and anti-arthritic activity of *Azima tetracantha* (LAM) whole plant in albino wistar rats**

Sridharan C^1^, Alvin jose M^1^, Radhakrishnan R^1^, Manisenthilkumar KT^2^

**^1^Swamy Vivekanandha College of pharmacy, Tiruchengodu, Tamilnadu; ^2^K.M.C.H. College of Pharmacy, Coimbatore, Tamilnadu, India.**

**Introduction:**

*Azima tetracantha* traditionally used as Antiinflammatory, antifungal, antibacterial also used for arthritic and toothache. This study is to evaluate the anti-arthritic activity of *Azima tetracantha* ethanolic extract (AZEE) in albino wistar rats.

**Method:**

Acute anti-inflammatory activity was evaluated by Carragenan induced paw edema method using diclofenac as standard. Anti-arthritis activity was evaluated by Freund’s Complete Adjuvant (FCA) induced arthritis. Wistar rats of both sexes weighing about 200-250gm divided into 5 groups of 6 each. AZEE 250-500mg was administred for 12days using dexamethasone as standard. The degree of inflammation was measured by Screw guage on 0, 1, 2, 3, 6, 9, 13, 15, 18 and 21days. On 21^st^ day blood samples were collected from all groups for evaluation of Rheumatoid factor and C-reactive protein.

**Results:**

AZEE 250-500mg/kg suppresses the Carragenan induced paw edema significantly at 3 to 4hrs (*P*<0.05, *P*<0.01). It’s a “BIPHASIC” response; AZEE 250/500mg significantly suppress paw edema in FCA induced rats (*P*<0.05, *P*>0.01). The odema in non-injected paw also suppressed by AZEE during 11^th^ and 16^th^ day. The body weight also improved with AZEE treated group compared with FCA group (*P*<0.01). Rheumatoid factor and C-reactive protein levels are decreased compared with FCA group (*P*<0.001).

**Conclusion:**

From the above findings we can conclude that AZEE has anti-arthritic, anti-inflammatory activity and it’s confirms the traditional claims. Thus needs further studies to prove the exact mechanism of action.

**165**

**Acute oral toxicity study of Rumipro™ in albino wistar rats**

Joshua AJ, Goudar KS, A Amit

**RandD Centre, Natural Remedies Pvt. Ltd., Bangalore, India.**

Rumipro, an herbal animal feed supplement for enhancing ruminal functions, was evaluated for its acute oral toxicity by administering as a single oral dose to female albino Wistar rats. Rumipro™ was administered orally in a sequential manner to five female rats at the limit dose level of 5000 mg/kg body weight. On the day of dosing, all the animals were observed for mortality and clinical signs for first 10 min, 30 min, 1 h, 2 h, 4 h and 6 h after dosing and thereafter twice daily for mortality and once a day for clinical signs, for 14 days. The body weight of rats was recorded and weekly body weight gain was calculated. After the observation period of 14 days, all surviving animals were sacrificed and subjected to complete necropsy. The Rumipro™ treated rats did not show any adverse clinical signs immediately following dosing and during the observation period of 14 days. Treatment with Rumipro™ did not reveal any major adverse effect on the body weight gain, except for three treated animals showing reduced body weight gain during the second week of 14 day observation period as compared to first week. Overall, the percent body weight gain during the complete 14 day observation period was found to be normal in all the treated animals. On necropsy, no major gross pathological changes were observed in any of the treated rats. Based on the findings of the present study, Rumipro™ was found to be safe after oral administration as a single dose to female albino Wistar rats up to 5000 mg/kg body weight.

**166**

**Evaluation of antidiabetic activity of methanolic extract of *Psoralea corylifolia* seeds in alloxaninduced hyperglycemic rats**

Rajak D^1^, Irchhaiya R^1^, Wanjari MM^2^

**^1^Institute of Pharmacy, Bundelkhand University, Jhansi, India;**

**^2^Central Research Institute (Ayurveda), Gwalior; India.**

**Introduction:**

The seeds of *Psoralea corylifolia* are indicated for treatment of diabetes in Ayurvedic system of medicine. Hence, it is proposed to evaluate antidiabetic activity of methanolic extract of *Psoralea corylifolia* seeds in alloxan-induced hyperglycemic rats.

**Materials and Methods:**

The methanolic extract of *Psoralea corylifolia* seeds (PCSE) was subjected to phytochemical screening and acute toxicity study. Hyperglycemia was induced in albino Wistar rats by single intravenous administration of alloxan monohydrate (65 mg/kg). Graded doses of PCSE (100, 200, 400 and 800mg/kg) suspended in carboxy methyl cellulose were administered orally to normal and diabetic rats, and to glucose loaded normal rats for oral glucose tolerance test (OGTT). Fasting serum glucose levels were assessed on different time intervals up to 3 h on first day and later on 3^rd^ and 7^th^ day of PCSE treatment. Metformin (500 mg/kg, orally) was used as standard antidiabetic agent.

**Results:**

PCSE was found to be safe up to 2000 mg/kg, orally (LD_50_>2000 mg/kg). PCSE treatment caused a significant dose- and time- dependant reduction in serum glucose levels in diabetic rats but not in normal rats. Similar effect was observed with metformin. In OGTT, PCSE suppressed elevated serum glucose levels in normal rats as did metformin.

**Conclusion:**

PCSE possesses antidiabetic effect. This effect of PCSE may be attributed at least in part to increased glucose metabolism as like other antihyperglycemic plant extracts.

**167**

**Pharmacological evaluation of hepatoprotective activity of roots of *Boerhaavia diffusa* and potentiation with piperine**

More YP, D’souza LL, Desai SK, Naik AB, Gawali VS

**Principal K.M.Kundnani College of Pharmacy., Mumbai, India.**

**Introduction:**

Despite scientific advances in our understanding of hepatotoxicity, and leads provided by traditional system of medicine, we do not yet have any effective entities to cure liver derangement, more importantly those which are caused by a variety of drugs. *Boerhaavia diffusa* has an established hepatoprotective activity, so such a plant will be easier to evaluate for the potentiating effect if any by piperine.

**Materials and Methods:**

Dried roots of *Boerhaavia diffusa* were extracted with 70% ethanol using Soxhlet apparatus. Hepatoprotective activity and subsequent evaluation for potentiation by piperine were carried out by administrating the ethanolic extract p.o. to albino wistar rats as per OECD-423 guidelines. The activity of *Boerhaavia diffusa* was assessed at two dose levels viz. 150mg/kg and 300 mg/kg. Piperine was used at two doses 10mg and 20mg. Two models were used to evaluate the hepatoprotective activity of *Boerhaavia diffusa*- (i) CCl_4_ induced hepatotoxicity and (ii) Rifampicin-Isoniazid induced hepatotoxicity. The biochemical parameters estimated for both models were the serum lysosomal enzymes SGOT, SGPT and ALP. Silymarin was used as a reference standard.

**Results:**

*Boerhaavia diffusa* showed a significant reduction in levels of the enzymes- SGOT, SGPT, ALP, which were elevated in the toxicant control groups thus showing good hepatoprotective activity. The evaluation of possible potentiation with piperine resulted in a dose dependent potentiation of the activity of *Boerhaavia diffusa*.

**Conclusion:**

The present study clearly demonstrates that *Boerhaavia diffusa* has good hepatoprotective activity in both animal models and its bioactivity is further enhanced in presence of piperine.

**168**

**Anti-inflammatory and anti-arthritic activity of a triterpenoid isolated from *Barringtonia racemosa***

Patil KR^1^, Patil CR^2^, Jadhav RB^2^, Surana SJ^2^, Fursule RA^1^

**^1^H. R. Patel Women’s College of Pharmacy, Shirpur, India; ^2^R. C. Patel Institute of Pharmaceutical Education and Research, Shirpur, India.**

**Introduction:**

*Barringtonia racemosa* has been reported to possess interesting biological potentials such as anti-tumor, anti-asthamatic, anti-inflammatory, analgesic, cytotoxic and anti-bacterial. There is a need to isolate the active components of this plant and screen them for their druggability.

**Methods:**

Isolation of triterpenoid: The targeted triterpenoid was isolated from *Barringtonia racemosa* by cold maceration of the powdered fruits of this plant in petroleum ether followed by extraction with methanol. The methanolic extract was fractionated with ethylacetate and was subjected to separation on a silica gel column with varying compositions of ethylacetate and chloroform. The isolation was monitored by TLC by comparing the isolated fractions with the Bartogenic acid as a marker.

**Characterization:**

The isolated triterpenoid was characterized for its chemical structure through HPTLC, IR, LC-MS and NMR analysis.

**Biological activity:**

The isolated compound was screened for its biological potentials through following assays;

1. Paw edema induced by Carrageenan, Histamine and serotonin in rats.
2. Granuloma induced by cotton pellet implantation in mice
3. Oxazolone induced ear edema in mice
4. Arthritis induced by Freund’s Complete Adjuvant in rats

Results and conclusion: The triterpenoid compound isolated by us matched with Bartogenic acid and was found to exert potent antiinflammatory and antiarthritic activity in the animal models. These activities were comparable with the standard drugs used in these study models. It is proposed that the Bartogenic acid is a major constituent responsible for biological potentials of *Barringtonia racemosa.*

**169**

**Hepatoprotective activity of Livosyp®, a polyherbal formulation: An *in vivo* study**

Srikanth I, Ghosh N, Singh PN, Kumar V

**Department of Pharmaceutics, Institute of Technology, Banaras Hindu University, Varanasi, India.**

**Introduction:**

Livosyp is a polyherbal formulation containing herbs which are known for their hepatoprotective properties in the Ayurvedic system of medicine. In this study, hepatoprotective activity of Livosyp was investigated.

**Methods:**

Hepatoprotective activity of Livosyp (at 100, 200 and 300 mg/kg, p.o.) was investigated in rats through validated models by inducing hepatotoxicity with carbon tetrachloride (CCl_4_): liquid paraffin (1:1) (2 ml/kg, i.p.) and paracetamol (250 mg/kg, p.o.). The degree of protection was measured by using biochemical parameters like serum transaminases (AST and ALT), alkaline phosphatase (ALP), bilirubin, hepatic malondialdehyde (MDA) concentrations and catalase and superoxide dismutase (SOD).

**Results:**

The single oral dose of paracetamol and CCl_4_ produced significantly elevated levels of serum AST, ALT, ALP, bilirubin activities. In addition, an increased MDA concentration and decreased catalase and SOD were observed in the hepatic tissues. Livosyp has been shown to lower the elevated serum levels of AST, ALT, bilirubin and alkaline phosphatase (ALP). Livosyp also significantly prevented the elevation of hepatic malondialdehyde formation in the liver of paracetamol and CCl_4_ intoxicated rats in a dose dependent manner. The decreased activities of catalase and SOD were also restored towards normalization by Livosyp administration. The results were confirmed histopathologically and compared with silymarin (100 mg/kg, p.o.), a standard hepatoprotective agent.

**Conclusion:**

The lower dose of Livosyp (100 mg/kg) did not produce significant hepatoprotective effect. However, 200 and 300 mg/kg doses of Livosyp have shown significant hepatoprotective activity compared to control while effect of 300 mg/kg dose of Livosyp was comparable qualitatively to silymarin.

**170**

**Opoid, adenosine, and 5HT_3_ receptors involvement in anti-nociceptive effect of ayruvedic polyherbal formulation RD/M.ph.AA-01**

Chandrasekar SB, Kalyani Divakar, Divakar Goli, L Prakash

**Acharya and B.M.Reddy College of Pharmacy, Bangalore-90, India.**

**Objective:**

For scientific validation of polyherbal formulation RD/M.ph.AA-01 evaluated for antinociceptive effect on animals in two different nociceptive induced models and also tested to reveal its mechanism of action.

**Methods:**

The effect was tested in wistar albino rats by tail immersion method (55±2°C) and acetic acid writhing test in swiss albino mice (20-30 gm) model.

**Results:**

The antinociceptive effect of RD/Mph-AA01 at the dose of 54 mg/200g.p.o. of rat was not significant but 81 mg/200g.p.o of rat was very significant (*P*<0.01) in tail immersion model. The percentage increase in reaction time after administration RD/ Mph.AA-01 (81mg/200g) was found to be 64.81, 78.14, 101.38, 67.12 and 27.31 at 30 min, 60 min, 120 min, 180 min and 240 min respectively. The peak antinociceptive effect was observed after 120 min of administration. The antinociceptive effect of RD/Mph. AA-01(81mg/200mg) was completely and significantly (*P*<0.01) abolished with prior administration of naloxone (2 mg/kg.i.p.).The prior administration of caffeine (16 mg/kg.i.p.) decreased maximum of 55.2% of antinociceptive effect of RD/Ph-AA01 (81mg/200mg) but stastically not significant. The antinociceptive effect of RD/Mph. AA-01 was significant (*P*<0.01) in writhing test. The percentage inhibition of writhes was 77.71 and 85.7 with RD/Mph.AA-01 at the dose of 7.8mg/20g and 11.7 mg/20g (p.o) respectively. The antinociceptive effect of RD/Mph.AA-01 (11.7 mg/20g) was completely and significantly (*P*<0.01) abolished with prior administration of 5-HT_3_ receptor antagonist ondosetron (0.5mg/ kg.i.p).

**Conclusion:**

RD/Mph.AA-01 has antinociceptive effect. Opioid, adenosine A1, A2 and 5-HT_3_ receptors are involved in its antinociceptive effect and inhibition of cytokine production might also account for its effect.

**171**

**Hypoglycemic activity of Indian *Hypericum perforatum* L. on alloxan-induced diabetic rats**

Husain GM, Singh PN, Kumar V

**Pharmacology Research Laboratory, Department of Pharmaceutics, Institute of Technology, Banaras Hindu University, Varanasi-221 005, India.**

**Introduction:**

Ethnobotanical knowledge played a particularly important role in historical diabetes therapies, with over 1200 species of medicinal plants recognized throughout the world for their ability to treat diabetic indications. *Hypericum perforatum* contains a variety of biologically active constituents including flavonoids like quercetin which have proved antidaibetic potential. Keeping this background in mind it seems realistic to evaluate Indian *Hypericum perfortatum* (IHp) for the control of diabetes.

**Methods:**

Charles foster rats of either sex (~180 g) were used in the present study. Hypoglycemic activity of the standardized extract of IHp (100 and 200 mg/kg) was observed on alloxan induced (120 mg/kg, i.p.) diabetic rats. IHp extract was orally administered as suspension in 0.3 % carboxymethylcellulose for 14 days. Glibenclamide (10 mg/kg/ day, p.o.) was used as standard. Blood samples were collected at 0, 7^th^ and 14^th^ day (1 hour after last dose) from orbital sinus and blood glucose was estimated by commercially available kit. Statistical analyses were performed by one way analysis of variance (ANOVA) followed by Dunnett Multiple Comparisons Test.

**Results and Conclusion:**

IHp led to significant fall (*P*<0.01) in elevated blood glucose level. Moreover, IHp treatment also reverses the weight loss associated with alloxan treatment. The mechanism by which this extract lowers blood glucose is under investigation. The observed blood glucose lowering effect suggests that IHp extract possess active phytochemical principles with hypoglycemic potential.

**172**

**Wound healing properties of *Kaempheria galanga* on wister rat**

Sharma C^1^, Shanbhag TV^2^, Adiga S^2^

**^1^Sikkim Manipal Institute of Medical Sciences; ^2^Kasturba Medical College, Manipal, Karnataka, India.**

**Introduction:**

Wound is the breach in the normal tissue continuum, resulting in variety of cellular and molecular scquelae. It can occur as a result of physical, chemical, thermal, electrical, radiational, surgical insult to tissue. Though the number of drugs ranging from the simple analgesic to complex and expensive chemotherapeutic agent administered for wound healing, a large number of world population are inaccessible to such drugs. A large section of population realized on the traditional medicine.

**Methods:**

During the present investigation, the plant *K. galanga*, which is widely used medicine in ayurvedic system of medicine, was taken for wound healing properties in Wistar Rat. Three type of wound model (incision, excision and dead space were employed to address all event of the wound healing process.

**Results:**

The study revealed that the rhizome of *K. galanga* facilitated epithelialization and reversed the dexamethasone suppressed healing, enhanced wound contraction, increased dry weight of the granulated tissue but did not alter the breaking strength of incision and dead space wound and hydroxyproline content.

**Conclusion:**

This study shows that the alcoholic extract of rhizomes of K. galanga mainly reverses the dexamethasone suppressed wound healing. This effect can be clinically made useful when healing has been adversely affected especially by drugs like antineoplastics, steroids, NSAIDS in perisurgical period and also to promote healing of leg ulcers, extensive surface burns, healing of donar area in skin graft surgery etc. These suggestions need well designed further clinical evaluation.

**173**

***In-vitro* antioxidant activity of alcoholic and aqueous extracts of *Achyranthes aspera* (asclepiadaceae)**

Modi HK, Patel NJ, Patel N

**S.K. Patel College of Pharmaceutical Education and Research, Mehsana, India.**

**Introduction:**

Free radicals are implicated for more than 80 diseases including diabetes mellitus, arthritis, cancer, liver diseases, ageing, conditions in which oxidative stress is prominent. In Indian system of medicine *Achyranthes aspera* is an important medicinal plant and its leaf, seed paste or root juice has been used in various ailments and as health tonic.

**Methods:**

To understand the mechanisms of pharmacological actions, the in-vitro antioxidant activity of alcoholic and aqueous extracts of leaf, root and seeds of *Achyranthes aspera* (ALLA, ALRA, ALSA, AQLA, AQRA and AQSA) was investigated for activity of DPPH free radicals, superoxide anion radicals and nitric oxide radicals scavenging assay spectrophotometrically. Ascorbic acid was taken as reference. Total phenolic contents in all extracts were determined by Folin Ciocalteu reagent.

**Result:**

The highest free radial scavenging activity was found with AQSA. IC_50_ values of AQSA was found 112.19 ± 2.21, 137.73 ± 2.58, 148.94 ± 3.95 mcg ml^-1^ in DPPH free radical, superoxide anion radical and nitric oxide radical assay respectively. The antioxidant activity of ALLA, AQLA was also found statistically significant. The total phenolic contents of AQSA, ALSA and AQSA were found 488.21, 386.54 and 413.28 respectively.

**Conclusion:**

From the results, we infer that antioxidant property of AQSA, ALSA and AQLA may be due to the presence of total phaenolic contents in extracts. These results clearly indicate that *Achyranthes aspera* is effective against free radical mediated diseases.

**174**

**Protective effects of aqueous extract of *Mucuna pruriens* linn. (DC) seed against gentamicin induced nephrotoxicity and oxidative stress in rats**

Modi KP^1^, Patel NM^1^, Goyal RK^2^

**^1^Shri B. M. Shah College of Pharmaceutical Education and Research, Modasa, India; ^2^L.M.College of Pharmacy, Ahmedabad, India.**

In the present study, we investigated the effects of aqueous extract of *Mucuna pruriens* on gentamicin (100 mg kg^-1^ per day for six days) induced nephrotoxicity and oxidative stress in rats. GEN-induced nephrotoxicity was evidenced by marked increase in serum urea and creatinine levels and urine volume and decrease in urinary sodium levels and creatinine clearance. *M. pruriens* extract at a dose of 200 mg/kg and 400 mg/kg in addition to gentamicin caused dose dependant decrease in serum creatinine and urea levels and an increase in creatinine clearance. There was a significant increase in the urinary sodium levels by *M. pruriens* extract at a dose of 400 mg/kg but not at the dose of 200 mg/ kg when compared with GEN control rats. Treatment with *M. pruriens* extract at a dose of 400 mg mg/kg further increased the urine volume as compared to GEN control rats. However, further increase in urine volume by *M. pruriens* extract at a dose of 200 mg/kg was not significant. Renal oxidative stress was determined by renal lipid peroxides levels, glutathione (GSH) levels and by enzymatic activity of superoxide dismutase (SOD) and catalase (CAT). GEN caused significant increase in lipid peroxide levels and significant decrease in GSH levels and SOD and CAT activity. *M. pruriens* extract significantly and dose-dependently reduced lipid peroxidation and enhanced GSH levels and SOD and CAT activities. Our data suggest that *M. pruriens* extract has protective potential against gentamicin induced nephrotoxicity and oxidative stress in rats.

**175**

**Evaluation of anti epileptic activity on methanolic leaf extract of *Passiflora foetida***

Ramaprasad P, Chan Basha S K, Santosh, Tirupati, Srikanth

**Vishnu college of Pharmacy, India.**

**Objective:**

To evaluate the anti epileptic activity of methnolic leaf extract of *Passiflora foetida*.

**Materials and Methods:**

Convulsions are induced by maximal electro shock(MES) in wistar rats. The drug *Passiflora foetida* leaf was given in two different doses ie 200mg/ kg, 300mg/kg and phenytoin is used as standard drug in the dose of 25mg/kg. The test drug was administered orally and a time gap of 45 minutes to 1 hour was allowed before inducing the convulsions through MES. A current of 150 ma was applied for 0.2 second at external ear. Different stages of convulsions are noted.

**Results:**

The methanolic leaf extrat of *Passiflora foetida* was found to be effective against convulsions induced by electro convulsive meter. There was significant reduction in extensor phase.

**Conclusion:**

Results suggest that the two different doses of methanolic leaf extrat of *Passiflora foetida* 200mg/kg, and 300mg/kg, possess good therapeutic activity against epilepsy.

**176**

**Effect of methanolic extract of *Myristica fragrans* on isolated rat vas deferens**

Hardik AJ, Sateesh B, Savai J, Kunte S

**School of Pharmacy and Technology Management, NMIMS University, Mumbai, India.**

**Introduction:**

*Myristica fragrans* Houtt. (nutmeg) are used for aromatic, carminative, aphrodisiac, anticariogenic and antioxidant activity. In Unani medicine used in the management of male sexual disorders. The present study was undertaken to study the effect of methanolic extract of Myristica fragrans (MEM) on oral acute toxicity in albino mice (OECD, 423) and its effect on isolated vas deferens of male albino rat.

**Methods:**

MEM subjected to thin-layer chromatography (TLC) and plates were developed in a mixture of toluene and ethyl acetate (93:7). The color was developed with vanillin. sulfuric acid reagent and *Rf* values were determined. Albino mice (25-30 g) were used for acute toxicity study (OECD, 423). Male Albino wistar rat (350 g), anaesthetized with pentobarbital sodium. Vas deferens were dissected out. The epididymal segment was cut and cleared of surrounding connective tissue. The tissue was then mounted in a 20 ml organ bath containing Kreb’s solution. The dose response curve of adrenaline (0.5×10^-3^) and MEM (5×10^-2^) were recorded. The actions were inhibited by prazosin (0.4×10^-3^). The EC_50_ values were calculated.

**Results:**

TLC analysis shown the presence of saffrol, myristicin, eugenol and elemicin. In the oral acute toxicity, the administration of MEM did not elicit any mortality upto 2 g/ kg bw in mice (OECD 423). In isolated vas deferens experiment, MEM shown adrenaline like effect. Further the contractions were inhibited by prazosin.

**Conclusion:**

The acute oral toxicity study revealed that the extract is safe and thus provides a scientific rationale for the traditional use of nutmeg in the management of male sexual disorders.

**177**

**Evaluation of anti-urolithiatic activity of methanolic extract of *Hemidesmus indicus* in rats**

Patel SJ, Agarwal SS, Baraiya PR, Patel JA

**Institute of Pharmacy, Nirma University of Science and Technology, Ahmedabad, India.**

**Objective:**

To evaluate the Anti-urolithiatic activity of methanolic extract of *Hemidesmus indicus* in rats.

**Introduction:**

*Hemidesmus indicus* belongs to family Asclepadiaceae. It is commonly known as Indian sarsaparilla or anantmul. It is found in many parts of India, from upper gangetic plain eastwards to Assam and throughout central, western and southern India.

**Materials and Methods:**

Methanolic extract of *Hemidesmus indicus* was administered orally at dose of 100, 200 and 400 mg/kg/day to healthy rats for 28 days. Cystone, an ayurvedic polyformulation, was used as standard drug and given at dose of 750 mg/kg/day. Urolithiasis was induced by two models. one was ethylene glycol induced urolithiasis where 0.75% ethylene glycol in drinking water was given for 28 days. And another model was uracil induced urolithiasis where 3% uracil in diet was given for 28 days. The assessment of anti-urolithiatic activity was done by measuring calcium, oxalate, phosphorous, uric acid, creatinine, sodium, potassium, Blood Urea Nitrogen, magnesium and citrate levels in urine, serum and kidney homogenate.

**Result:**

The methanolic extract of *Hemidesmus indicus* produced dose dependent decrease in calcium, oxalate, phosphorous, uric acid, creatinine, Blood Urea Nitrogen levels in urine, serum and kidney homogenate and the anti-urolithiatic activity of *Hemidesmus indicus* was comparable to the cystone.

**Discussion and Conclusion:**

oral administration of the methanolic extract of *Hemidesmus indicus*, in present study produced significant anti-urolithiatic activity. Proper formulation of above indigenous drug may prove to be a promising therapy for the treatment of renal stones in clinical practice.

**178**

**Hepatoprotective activity of aqueous-alcoholic extract of *Pedalium murex* (bada gokhru) in ethanol and isoniazide hepatotoxic rats**

Ladani K, Patel N J, Patel N, Solanki A

**S K Patel College of Pharmaceutical Education and Research, Kherva-382711, North Gujarat, India.**

The role of oxidative stress and ROS generation has been proved in the pathogenesis of liver damage. ROS initiate auto oxidation of cellular membrane lipids can lead to cellular necrosis thus they are well known to be cytotoxic and have been implicated in the etiology of hepatotoxicity and possibly represent the hepatotoxic principle of alcohol and various drugs. In this present study, we have assessed acute oral toxicity of aqueous-alcoholic extract of fruits of *Pedalium murex* on female Swiss albino mice by AOT 425 guideline. Hepatoprotective activity of aqueous-alcoholic extract of fruits of *Pedalium murex* (200, 400 mg/kg) was carried out using alcohol (40% ethanol) and isoniazide induced liver damage in wistar albino female rats. In same models, we have measured free radical scavenger enzymes (SOD, Catalase) level. Aqueousalcoholic extract of fruits of *Pedalium murex* did not show any mortality up to 5000 mg/kg. Significant hepatoprotective activity at 400 mg/kg dose of aqueous-alcoholic extract was observed in alcohol and isoniazide induced liver damage. All the elevated liver biochemical parameters (SGPT, SGOT, TB, TG and TC) in alcohol and isoniazide intoxicated rats decreased significantly near to normal level by aqueous-alcoholic extract of fruits of *Pedalium murex*. Photomicrograph of liver sections also has shown hepatoprotective activity. SOD and Glutathione level was significantly increased and Lipid peroxidation was significantly reduced with 400 mg/kg dose of aqueous-alcoholic extract of fruits of *Pedalium murex*. Flavonoids and tannins present in aqueousalcoholic extract may responsible for hepatoprotective action by scavenging free radicals.

**179**

**Lipid lowering effect of *Moringa oleifera* in poloxamer-407 and high fat diet induced hyperlipidemia**

Rajanandh MG, Satish Kumar MN, Kiran C, Elango K, Vadivalen R, Shanish, Antony A, Sethi N, Das A, B Suresh

**J.S.S college of pharmacy, Ooty, India.**

The leaves of *Moringa oleifera* Lam (Moringaceae) have anti-diabetic, anti-tumor and used for the treatment of cardio vascular and liver diseases by traditional healers in India. The several published reports support the same. The present study investigated the lipid lowering effect of the leaves of plant *Moringa oleifera* in poloxamer- 407 (1g/kg/b.wt) and high fat diet induced hyperlipidemic rats. The experimental animals were divided into five groups of male Wistar rats each. The results indicate that oral administration of hydro alcoholic extract of *Moringa oleifera* (HEMO) at two different dose level (100 and 200 mg/kg/b.wt) showed significant (*P*<0.001) reduction in elevated levels of total cholesterol, triglycerides, low density lipoproteins, very low density lipoproteins and similarly significant (*P*<0.001) increase in high density lipoproteins level in both poloxamer-407 and high fat diet induced hyperlipidemic rats. Atherogenic index was significantly reduced in the *Moringa oleifera* treated groups at tested dose levels. Histopathology reports of liver and aorta evidence the similar finding. The quantitative analysis of HEMO by using LC-MS indicated the presence of 0.09% β-sitosterol. It suggests that β-sitosterol is a bioactive phyto principle in the leaves of *Moringa oleifera*, may be responsible for its lipid lowering effect and it can be prescribed as food supplement for coronary artery disease patients along with their regular lipid lowering therapy.

**180**

**Effect of prophylactic treatment of extract of *Cichorium intybus* Linn. against acetaminophen induced hepatocellular damage in albino mice**

Tabassum N^1^, Shah MY^2^, Qazi MA^3^, Shah A^4^

**^1,2^Deptt. of Pharmaceutical Sciences, University of Kashmir, Srinagar, India; ^3^Deptt. of Pharmacology, G.M.C, Srinagar JandK, India; ^4^Deptt. of Pathology, SKIMS, Soura, Srinagar, India.**

**Introduction:**

These days in the developed as well as developing countries, herbal medicines are in great demand because of their wide biological activities, higher safety margin and lesser costs than the synthetic drugs. *Cichorium intybus* Linn.(Kashni) has been advocated in traditional system of medicine for the treatment of liver disorders. In the present study, extract of Kasni was evaluated for its prophylactic activity against acute hepatocellular damage induced in albino mice.

**Materials and Methods:**

Ethanolic extract of Kashni was administered at three dose levels (50, 100 and 200mg/100g/ day) for five days to albino mice. Hepatic damage was induced by acetaminophen (500mg/kg, single dose). Serum ALT levels were estimated biochemically and liver sections were subjected to histopathological studies.

**Result:**

Significant rise (***P<0.001) was observed in serum ALT levels in mice after administration of acetaminophen. Extract of Kashni, when administered in different doses prophylactically for five days before administration of acetaminophen, produced a dose dependent fall in the enzyme levels, the decrease being significant (*P*<0.05and *P*<0.001) with 100 and 200mg/100g doses, respectively. Histopathological studies revealed marked regenerative activity in the livers at the three doses of the extract.

**Conclusion:**

It is concluded that *Cichorium intybus* has significant prophylactic antihepatotoxic activity as it lowers the ALT levels in mice in which hepatotocellular damage was induced by acetaminophen and increases the regenerative activity of liver cells.

**181**

**Antinociceptive activity of *Terminalia arjuna* in mice**

Halder S, Bharal N, Mediratta PK, Sharma KK

**University College of Medical Sciences, Delhi, India.**

**Introduction:**

*Terminalia arjuna (T. arjuna)*, an Indian Medicinal Plant, has been known for the treatment of angina and other cardiac ailments. This study investigates the effect of *T. arjuna* in animal models of nociception.

**Methods:**

The antinociceptive activity was determined by tail flick latency test and formalin –induced pain response in mice. *T.arjuna* was administered orally in doses of 400 and 800mg/kg, p.o 1hr before the nociceptive stimulus. In a separate series of experiments, mice were pretreated with morphine for three days in increasing doses (10, 15, 20mg/kg, i.p.), and the antinociceptive responses to morphine (5mg/kg, i.p.) and *T. arjuna* (800mg/kg, p.o.) were observed in morphine tolerant animals.

**Results:**

*T. arjuna* (400mg/kg, p.o.) significantly reduced the duration of licks and bites in both phases of formalin-induced pain response. Tail flick latency test showed significant increase at higher dose of *T.arjuna* (800mg/kg, p.o.).These effects were antagonised by pre-treatment with naloxone..In morphine tolerant mice, cross tolerance was observed with T.arjuna (800mg/kg, p.o.) administration.

**Conclusion:**

The effect appears to be mediated by opoidergic mechanism as the antinociceptive effect of *T.arjuna* was antagonised by naloxone and there was cross tolerance to *T.arjuna,* in morphine tolerant mice. Thus *T. arjuna* can be said to possess antinociceptive potential.

**182**

**Anticonvulsant activity of milk extract of nuts of *Sememcarpus anacardium*, linn**

Shaikh MF^1^, Kenjale R^1^, Shah D^1^, Sathaye S^1^, Rao NV^2^

**^1^University Institute of Chemical Technology, Mumbai, India; ^2^V. L. College of Pharmacy, Raichur, India.**

*Semecarpus anacardium,* Linn. is reported with varied important activities in traditional system of medicine. In the present work, the nuts of *S. anacardium* extracted with milk (MENSA) were studied for its anticonvulsant potential using Maximal electroshock (MES) model. The nut extract of *S. anacardium* was prepared by following Siddha method of extraction. Two dose levels of MENSA were used. Seizures were induced in rats by delivering electroshock of 150mA for 0.2 s by means of electroconvulsiometer. Each animal was placed into individual plexiglass transparent cage and was observed for 30min. Time (seconds) in various phases of convulsions was noted. A decrease in duration of hindlimb extension indicates anticonvulsant activity. All the experimental groups were compared with the respective control treated with vehicle. MENSA was found to posses anticonvulsant activity against MES-induced convulsions and hence can be used in both tonic and tonic-clonic type of seizures.

**183**

**Diuretic activity of roots extract of *Rubia cordifolia* L**

Pawar AT, Kalyani Divakar, Goli Divakar, Sahu R

**Acharya and B. M. Reddy college of Pharmacy, Bangalore, India.**

**Introduction:**

*Rubia cordifolia* Linn. (family: Rubiaceae) is known as Indian madder. Roots are traditionally used as anti-inflammatory, astringent, antiseptic, antidysentric and blood purifier. The present investigation was carried out to evaluate the diuretic activity of the hydroalcoholic extract of roots of *Rubia cordifolia* (HARC) in rats.

**Methods:**

Roots of *Rubia cordifolia* was coarsely powdered and extracted using 70% ethanol with soxhlet extractor for 22h. Animals were treated with two doses of extract (HARC-286 mg/ kg, HARC-667 mg/kg bw) or vehicle or furosemide (20 mg/kg) orally. Urine excreted was collected up to 5h post-treatment and analyzed for urine volume, Na^+^, K^+^, Cl^−^ and creatinine.

**Results:**

The extract showed a significant (*P*<0.01) and dose dependent increase in urine volume and electrolyte excretion. The volume of urine excreted was 1.97±0.04 and 3.33±0.12 ml/animal by 286 and 667 mg/kg of HARC treated animals respectively, whereas 0.87±0.03 and 6.60±0.24 ml/animal by vehicle and furosemide treated animals respectively. The sodium level in urine (meq/5h/animal) was 0.09±0.004, 1.29±0.047, 0.36±0.009 and 0.62±0.020 in vehicle, furosemide, 286 and 667 mg/kg of HARC treated animals respectively. The potassium level in urine (meq/5h/animal) was 0.07±0.002, 0.31±0.002, 0.12± 0.002 and 0.23±0.011 by animals treated with vehicle, furosemide, 286 and 667 mg/kg of HARC respectively, whereas chloride level in urine (meq/5h/animal) was 0.13±0.004, 1.48±0.054, 0.33±0.013, 0.64±0.022 by vehicle, furosemide, 286 and 667 mg/kg of HARC treated animals respectively. Both doses of HARC showed less influence on creatinine clearance than furosemide.

**Conclusion:**

The result indicates that hydroalcoholic extract of roots of *Rubia cordifolia* possess diuretic activity.

**184**

**Iron chelation activity of young leaf juice of barley (*Hordeum vulgare* L.)**

Samant GG, Jadhav SR, Kshatriya AA, Sancheti J S, Saraf MN

**Bombay college of Pharmacy, Mumbai, India.**

**Introduction:**

Iron chelation therapy is used in the treatment of iron overload disease like thalassemia. Polyphenols baring catechol or galloyl moiety form a stable chelate with iron. Saponarin and lutonarin are the major flavonoids (polyphenols) found in barley leaves (*Hordeum vulgare* L.) and as lutonarin contains the catechol moiety *in vitro* and *in vivo* studies were performed to investigate the iron chelation activity of Barley leaf juice (BL).

**Methods:**

*In vitro:*Determination of % iron (II) chelating activities of BL. *In vivo:* Female SD rats (n=5) were administered with BL (perorally) in three different doses (1, 3 and 5 gm/kg) and Deferiprone used as standard (100 mg/kg) for 8 days. Animals were acutely loaded with iron (125 mg/kg) on 6^th^day. The plasma free iron concentrations were measured on 8^th^day.

**Results:**

*In vitro:* The % chelating activities of 20 mg/ml, 30 mg/ml, 40 mg/ml, 50 mg/ml, 60 mg/ml and 70 mg/ml solutions of BL were found to be 32.75%, 49.94%, 60.82%, 72.97%, 73.92% and 86.96% respectively. *In vivo:* The free iron levels of plasma were reduced in test groups in dose dependent manner. On 8^th^ day free iron concentrations in plasma of standard and all 3 test groups were reduced to 55.56 %, 85.41%, 82.35% and 72.01% respectively as compared to control group.

**Conclusion:**

This investigation revealed that BL can chelate iron effectively. The results indicate the potential of BL in the treatment of iron overload diseases like Thalassemia, Sickle cell anemia.

**185**

**Crude drugs and their use in the traditional medicine practices in western Nepal**

Kaundinnyayana A^1, 2^, Banstola A^2^, Shrestha H^2^, Karki SJ^2^, Panta S^2^, Koirala RR^3^

**^1^Svaddhyayashala, Kathmandu Nepal: ^2^Pokhara University, Pokhara, Nepal; Ayurveda Health Home, Kathmandu, Nepal.**

Traditional Medicine (TM) is widely followed throughout the world especially in Asian and African countries. In Nepal apart from the ethnic folk medicine, Ayurvedic medicine and Tibetan medicine have rich heritage of over many centuries and major population is still relying on them. These systems use crude products of plants, animals and minerals as medication. In the present work, we have studied the traditional systems of medicines practiced in the western Nepal according to the crude drugs used and their formulations. For this firstly using the Knowledge Attitude and Practice (KAP) survey method, we studied the status and popularity of the traditional medicines in comparison to the modern medicine in the selected villages of our study area which lie in the temperate and sub-alpine region of western Nepal. Then the short listed crude drugs which included mainly the Medicinal and Aromatic Plants (MAPs) were collected, identified and preserved. Alongside we documented the indigenous knowledge obtained from the locals including the traditional medical practitioners *(Baidyas, Amchis, Lamas Dhamis* and *Jhankris)* and the tribal elders regarding the use of the crude drugs. More than hundred crude drugs and over fifty specific formulations were documented. We believe that the study would help in the scientific exploration of the traditional knowledge. Dissemination of thus achieved knowledge, discussion on the conservation, appropriate use and the possibility of the discovery of potential new drugs from the crude drugs studied can also be achieved and indicated from this work.

**186**

**Microbiological profile, safety evaluation and anti microbial effects of *Cinnamomum zeylanicum***

Najmi AK^1^, Alhindi RR^2^

**^1^Jamia Hamdard, New Delhi, India; ^2^King Abdul Aziz University, Jeddah, Saudi Arabia.**

Samples of the bark of *Cinnamomum zeylanicum* were evaluated to generate data about its microbiological profile and antimicrobial effects. 15 samples of cinnamon were procured from different regions of Jeddah province, Saudi Arabia (SA). These samples were evaluated for their microbiological qualities i.e. contamination load of total aerobic mesophilic bacteria (TAMB), mesophilic aerobic sporeformer bacteria (MASB), coliform bacteria (CFB), and lactic acid bacteria (LAB), aflatoxins (B1, B2, G1, G2 and Ochratoxin A) and pesticides residue, food borne bacterial pathogens such as *Bacillus cereus, Staphylococcus aureus, Clostridium perfringes,* The samples were also evaluated for antimicrobial activity against *Listeria monocytogenes* and *Escherichia coli* 0157:H7. All the samples of cinnamon were found to be having some load of TAMB and MASB in the range of log 4 to log 5 cfu/gm but free from CFB and LAB. Samples were also contaminated with yeast, mold and *Clostridium perfringes* but free from coagulase (+) staphylococcus and *Bacillus cereus.* Extract of cinnamon showed inhibition against the *Listeria monocytogenes* and *Escherichia coli* 0157:H7 even after 2 hours of incubation period by a log factor of 4.

**187**

**Suppression of inflammation and cartilage destruction by hydroalcoholic extract of *Calotropis procera***

Parihar G^1^, Srivastava DN^2^

**^1^COP IPS Academy, Indore, India, ^2^B.R. Nahata college of Pharmacy, Mandsaur, India.**

**Objective:**

To study the effect of *Calotropis procera* on Freund’s complete adjuvant induced arthritic rats.

**Materials and Methods:**

Arthritis was induced by administering 0.1 ml of Freund’s complete adjuvant (FCA) in adult male. Arthritic animals were treated with hydroalcoholic extract of *calotropis procera* (100 / 200 mg/kg) orally from day 14 to day 28 after immunization. Parameters such as clinical parameter arthritic score paw volume, radiological examinations of right hind paw, determination of oxidative stress parameters lipid peroxidation (TBARS), Reduced Glutathione (GSH) in liver, and total plasma thiols content, histolopathology of hind paw were evaluated.

**Result:**

Hydroalcholic extract of *Calotropis procera* significantly (*P*<0.05) reduce inflammation as shown by reduction in arthritic score and paw volume. No secondary signs of arthritis were seen. Oxidative stress associated with FCA-induced arthritis was ameliorated evaluated by measuring the levels of GSH, TBARS content in the liver and total plasma thiol. The radiological and histological pictures of the joints arthritic animals show the amelioration of inflammation in joints of drug treated arthritic animals projecting the bones of ankle joint with intact articular cartilage and no inflammatory cells are present. Bones of ankle joint of drug control animals with no evidence of inflammation and inflammatory cells. Dexamethasone was more effective in inhibiting paw inflammation as compared to test extract.

**Conclusion:**

Thus, present study shows that the roots of *Calotropis procera* markedly reduce cell influx, inflammation, and oxidative stress associated with arthritic condition, and therefore has the potential to be used as an antiarthritic agent.

**188**

**Analgesic and anti-inflammatory of *Anthocephalus cadamba* leaves**

Buchke VA, Bachhav RS, Saudagar RB

**Department of Pharmacology, Pharmacognosy and Pharmaceutical Chemistry KCT’s RGS College of Pharmacy, Nashik, Maharashtra, India.**

**Objective:**

To study the analgesic and anti-inflammatory activity of a aqueous extract (AE) *Anthocephalus cadamba* leaves.

**Materials and Methods:**

Male albino mice (20-25 gm), male wistar rat (100-150gm) were obtained. Animals were housed in to groups of five at an ambient temp. of 25±1°c. Animals had free access to food and water. Animals were deprived of food but not water 4 h before the experiment. Aspirin (20 mg/kg p.o.), Carrageenan (0.1 ml of 1% w/v sub plantar) these drugs were dissolved in 2% gum acacia solution before administration the aqueous extract (ME) was prepared by extraction process. The effect of aqueous extract (AE) of *Anthocephalus cadamba* (AC) (50, 100, 300, mg/kg, p.o.) was investigated for analgesic activity using acetic acid induced writhing, tail immersion method and hot plate method. For anti-inflammatory activity the aqueous extract (AE) of *Anthocephalus cadamba* (AC) leaves was studied (50, 100, 300, mg/kg p.o.) using carrageenan induced inflammation.

**Result:**

Aqueous extract of AC shows (50, 100, 300, mg/kg p.o.) significantly (*P*<0.05*) increased latency to in tail flick immersion method, significantly (*P*<0.05*) reduced the number of writhing induced by acetic acid and significantly (*P*<0.05*) elevated in mean basal reaction time in hot plate method. The AC (50, 100, 300, mg/kg p.o.) also shows significant (*P*<0.05*) anti-inflammatory activity.

**Conclusion:**

The aqueous extract (AE) of AC leaves posses analgesic and anti-inflammatory activity as dose dependent.

**189**

**Amelioration of oxidative stress and renal dysfunction by insulin and its combination with curcumin or resveratrol: Role of TGF-β**

Tiwari V^1^, Sharma S^1, 2^, Kulkarni SK^1^, Chopra K^1^

**^1^University Institute of Pharmaceutical Sciences, Panjab University, Chandigarh, ^2^New Drug Discovery Research, Ranbaxy Research Laboratories, Gurgaon, India.**

The aim of the present study was to examine the effect of insulin and its combination with resveratrol or curcumin on TGF-beta levels in experimental model of diabetic nephropathy. Diabetes was induced by a single intraperitoneal injection of STZ in rats. After 4 weeks of STZ injection, rats were divided into seven groups: the control rats, diabetic rats and diabetic rats treated with insulin (10 IU/kg/s.c) or resveratrol (20mg/kg/p.o.) or curcumin (60mg/kg/p.o.) per se and the combination of resveratrol or curcumin with insulin, from week four uptil week eight. At the termination of the experiments, the renal function tests, oxidative stress markers malonaldehyde and glutathione levels and the anti-oxidant enzymes superoxide dismutase and catalase were measured in kidney homogenate. STZ-injected rats showed significant increases in blood glucose, polyuria, proteinuria and a decrease in body weight compared with age-matched control rats. After 8 weeks, diabetic rats exhibited renal dysfunction along with a marked increase in oxidative stress, as determined by lipid peroxidation and activities of key antioxidant enzymes. Diabetic animals also exhibited a significant increase in TGF-beta levels. Chronic treatment with insulin and its combination with resveratrol and curcumin significantly attenuated renal dysfunction and oxidative stress in diabetic rats. There was a significant inhibition of TGF-beta release when these drugs were given in combination as compared to their effects per se. These results indicate an anti-oxidative mechanism of resveratrol and curcumin and point towards the beneficial effect of these combinations for their renoprotective action.

**190**

**Anti-oxidant activities of kiwi extract on CCl_4_- induced liver injury in mice**

Won young K, Heekyoung Y, Hyun Ju H, Sung Ho L, Yu Jin C, Tae Hyuk K, Jong Hwe K, Chang Hoon H, Young Jae L^1^

**Cheju National University, Jeju, Korea.**

The kiwi (Actinidia deliciosa) is well known for its anti-oxidant ingredient in it. In this study, we investigated the anti-oxidant effects of kiwi extracts on carbon tetrachloride (CCl4) induced liver injury in BALB/c mice. Radical scavenging effects of 80% methanol extracts of three kinds of kiwi, Halla-Gold, Zesi-sweet, and Zespri, were observed using 2, 2-diphenyl-1-picrylhydrazyl (DPPH) method, nitroblue tetrazolium (NBT) reduction method. In addition, inhibititory activity of extracts for lipid peroxidation was examined using thiobarbituric acid-reactive substances (TBARS) assay. For animal study, antioxidant potential of kiwi extracts were examined in BALB/c mice by inducing liver injury with carbon tetrachloride. Intraperitoneal administration of CCl4 resulted in significantly elevated plasma levels of alanine aminotrans ferase (ALT) and aspartate aminotransferase (AST) in control group. However, when mice were pretreated with the kiwi extracts, ALT and AST levels in plasma were decreased. In the liver, kiwi extracts had strong antioxidative activity in the MDA production and in the glutathione level. Moreover, anti-oxidant enzymes such as superoxide dismutase (SOD), catalase, glutathione peroxidase (GSH-px) and glutathione reductase were restored in kiwi extracts treatment groups. Histopathological features were restored in kiwi extracts treated groups compared with those of CCl4-induced hepatotoxicity group.

On the basis of the results obtained it can be concluded that the kiwi showed a certain protective effect through not only the antioxidant, but also the activities in CCl_4_-intoxicated mice.

**191**

**Effect of resveratrol on biochemical markers of insulin resistance**

Wadhvani PJ, More SM, Gandewar S, Chopde CT

**Smt. Kishoritai Bhoyar College of Pharmacy, Nagpur, India.**

**Introduction:**

Alterations in various biochemical constituents like glucose, triglycerides, cholesterol, HDL and adiponectin in blood of diabetic patients signifies the development of insulin resistance. Resveratrol, a natural phytoalexin abundantly found in red grapes, tomatoes and peanuts is beneficial in lipid metabolism and increases insulin secretion. In view of this, present study was designed to investigate the effect of chronic administration of resveratrol on biochemical changes associated with insulin resistance.

**Methods:**

Insulin resistance was induced in male Sprague Dawley rats by exposure to high fructose diet for 21 days followed by food deprivation for 24 hrs. Animals received the resveratrol (15 *μ*g/kg, i.p.) daily for 21 days. Blood samples were collected on 22^nd^ day by retro-orbital route and analyzed for glucose, triglycerides, cholesterol, HDL and adiponectin blood levels using the respective estimation kits.

**Results:**

Resveratrol treated rats showed a significant decrease in the blood glucose and triglycerides levels while it increased the HDL and adiponectin levels as compared to the fructose fed group. It also significantly reduced the body weight in high fructose fed rats compared to control rats.

**Conclusion:**

The present study demonstrates that increase in adiponectin levels by resveratrol treatment increases insulin sensitivity. At the same time, increased glucose and triglyceride levels which contribute to insulin resistance were also decreased significantly. In conclusion, the present study indicates resveratrol administration or consumption of resveratrol rich food may offer a better therapeutic approach for the treatment of insulin resistance.

**192**

**Evaluation of anti-tussive activity of herbal drugs in sulphur dioxide (SO_2_) induced cough model in mice**

Kumar G, Mehla J, Katyal J, Gupta YK

**Neuropharmacology Laboratory, Department of Pharmacology, All India Institute of Medical Sciences, New Delhi-29, India.**

**Background:**

Cough is the most common symptom of respiratory diseases. When cough becomes serious, opioids are effective, but they have side effects like sedation, constipation, some addiction liability and also compromise the respiratory function. Therefore, there is need to have effective anti-tussive agent which do not have respiratory suppressant activity. The present study was carried out to evaluate anti-tussive activity of combination of herbal drugs in sulphur dioxide (SO_2_) induced cough model in mice.

**Materials and Methods:**

The experiments were performed on mice of either sex, weighing 25-35g. The mice screened for number of cough bouts after exposure to SO _2_ in a dessicator and those showing 60+10 cough bouts in five minutes served as normal control. Mice were randomly divided in to 7 experimental groups (n=6). Group 1 as normal control, group 2 were given distilled water, group 3 as positive control received codeine sulphate (10mg/kg, p.o.) and group 4, 5, 6, 7 received coded herbal drugs I, II, III. IV respectively at a dose of 0.3ml/ mice, orally, using feeding canula and 20 minutes latter were exposed to sulphur dioxide again for 45 sec. The mice were then placed in an observation chamber for counting of cough bouts by two independent observers. Readings are mean of these two observations and from this percent inhibition in number of cough bouts was calculated.

**Results:**

In normal controls, there was no significant change in number of cough bouts between the two exposures while mice with distilled water showed a reduction of 20.5%. Codeine (10mg/kg, oral) pretreatment group suppressed cough bouts by 73.5% while herbal formulation code I, code II, code III and code IV suppressed the cough bouts by 71.5%, 49.5%, 58.8%, and 58.5% respectively.

**Conclusion:**

All the herbal formulations used showed significant antitussive activity in sulphur dioxide induced cough model. Thus, they can prove to be useful for alleviating cough.

